# Identification and Immunocorrelation of Prognosis-Related Genes Associated With Development of Muscle-Invasive Bladder Cancer

**DOI:** 10.3389/fmolb.2020.598599

**Published:** 2021-01-29

**Authors:** Jingxian Li, Yantao Lou, Shuai Li, Fei Sheng, Shuaibing Liu, E. Du, Zhihong Zhang

**Affiliations:** ^1^ Tianjin Institute of Urology, The Second Hospital of Tianjin Medical University, Tianjin, China; ^2^ Tianjin Hospital, The Tianjin Medical University, Tianjin, China

**Keywords:** muscle-invasive bladder cancer, weighted gene co-expression network analysis, prognosis-related genes, tumor immune infiltration, targeted therapy

## Abstract

Improved understanding of the molecular mechanisms and immunoregulation of muscle-invasive bladder cancer (MIBC) is essential to predict prognosis and develop new targets for therapies. In this study, we used the cancer genome atlas (TCGA) MIBC and GSE13507 datasets to explore the differential co-expression genes in MIBC comparing with adjacent non-carcinoma tissues. We firstly screened 106 signature genes by Weighted Gene Co-expression Network Analysis (WGCNA) and further identified 15 prognosis-related genes of MIBC using the univariate Cox progression analysis. Then we systematically analyzed the genetic alteration, molecular mechanism, and clinical relevance of these 15 genes. We found a different expression alteration of 15 genes in MIBC comparing with adjacent non-carcinoma tissues and normal tissues. Meanwhile, the biological functions and molecular mechanisms of them were also discrepant. Among these, we observed the ANLN was highly correlated with multiple cancer pathways, molecular function, and cell components, revealing ANLN may play a pivotal role in MIBC development. Next, we performed a consensus clustering of 15 prognosis-related genes; the results showed that the prognosis, immune infiltration status, stage, and grade of MIBC patients were significantly different in cluster1/2. We further identified eight-genes risk signatures using the least absolute shrinkage and selection operator (LASSO) regression analysis based on the expression values of 15 prognosis-related genes, and also found a significant difference in the prognosis, immune infiltration status, stage, grade, and age in high/low-risk cohort. Moreover, the expression of PD-1, PD-L1, and CTLA4 was significantly up-regulated in cluster1/high-risk-cohort than that in cluster2/low-risk-cohort. High normalized enrichment score of the Mitotic spindle, mTORC1, Complement, and Apical junction pathway suggested that they might be involved in the distinct tumor immune microenvironment (TIME) of cluster1/2 and high-/low-risk-cohort. Our study identified 15 prognosis-related genes of MIBC, provided a feasible stratification method to help for the future immunotherapy strategies of MIBC patients.

## Introduction

Bladder cancer has become common globally due to its prevalence, high recurrence risk, and treatment failures (Antoni et al., 2017; Martinez Rodriguez et al., 2017; Bhanvadia, 2018; Richters et al., 2020). The principal diagnosis of BLCA is non-muscle invasive bladder cancer (NMIBC), muscle-invasive bladder cancer (MIBC), and metastatic bladder cancer (Kamat et al., 2016). MIBC is defined by tumor invasion into the detrusor muscle, perivesical tissues, or neighboring organs. Although the treatment of MIBC has improved, the prognosis of MIBC patients with metastatic urothelial cancer is generally poor, with overall 5-years survival of 15% despite chemotherapy (Schneider et al., 2019). Therefore, better biomarkers for specific prognosis and progression of MIBC are necessary. Moreover, an improved insight of MIBC molecular mechanisms and immunoregulation might lead to identifying new targets for future therapies.

The development of genomic techniques provides a foundation for utilizing bioinformatics to explore the genome-wide characterization of diseases (Akalin, 2006). As a significant public health concern, the pivotal biomarkers of MIBC remain ambiguous. Bioinformatics provides a reference to screen the potential biomarkers of MIBC and predict the molecular mechanism and clinical relevance of these biomarkers. Combined analysis of different databases, such as TCGA and GEO, helps to elevate the discriminating ability of highly related genes of MIBC that are useful to serve as candidate biomarkers. Meanwhile, the combination of multi-bioinformatics methods, such as differential expression analysis and WGCNA, also elevate the precision of screened biomarkers of MIBC. Furthermore, emerging bioinformatics resources could assist in characterizing the MIBC immune microenvironment and revealing the correlation between biomarkers and immune infiltration.

In this study, we analyzed the mRNA expression of MIBC in the TCGA and GEO database using differential expression analysis and WGCNA. The univariate Cox progression analysis identified 15 MIBC prognosis-related genes. Further, we conducted a series of analyses, such as survival analysis, Protein-protein interaction (PPI), Gene Set Enrichment Analysis (GSEA), and Gene Set Variation Analysis (GSVA), to discover the molecular mechanism, clinical relevance, and cross-talk of these 15 genes. Finally, we established consensus clustering and risk model of these 15 genes, provided a potential stratification method to screen out MIBC patients who are sensitive to immunotherapy, and help for the future treatment strategies of these patients.

## Materials and Methods

### Datasets From TCGA, GTEX, and GEO Database

We downloaded the gene expression profiles of MIBC from the TCGA (https://xena.ucsc.edu/), GTEX (https://xena.ucsc.edu/), and GEO (https://www.ncbi.nlm.nih.gov/gds) database. The RNA-seq expression value of the TCGA and GTEX datasets were transformed to log2(FPKM + 1) unit to allow for subsequent analysis. There were 435 bladder samples, including 407 muscle-invasive bladder cancer tissues, 19 adjacent non-carcinoma tissues, and nine GTEX normal tissues. The GSE13507 dataset obtained from the GEO database was transformed to log2(count) and normalized using limma R-package. GSE13507 dataset consisted of 62 MIBC samples, 58 adjacent non-carcinoma tissues, and 10 normal tissues from the patients. We matched the probes to the gene symbols on the basis of a manufacturer-provided annotation file. The duplicated probes for the same gene were removed by determining the median expression value of all its corresponding probes.

### Differential Expression Analysis and Identification of Key Co-Expression Modules

We respectively screened the differential expression genes (DEGs) between MIBC and adjacent non-carcinoma tissues applying the limma R-package downloaded from the Bioconductor (https://www.bioconductor.org/) in the TCGABLCA and GSE13507 dataset. The *p*-value was adjusted by the Benjamini Hochberg method to control the false discovery rate (FDR). DEGs were filtered using the adjusted *p*-value <0.05 and |log_2_(Fold change)| > 1. 1,664 DEGs from TCGABLCA and 377 DEGs from the GSE13507 dataset were screened and then subjected to the WGCNA package to identify the key co-expression modules. The WGCNA was used to excavate the modules of highly correlated genes among samples for relating modules to external sample traits (Langfelder and Horvath, 2008). The Pearson analysis of the DEGs was performed to construct a matrix of similarity. According to the power value (*β* = 6 in TCGA-MIBC and 14 in GEO-MIBC), which mainly affects the independence and the average degree of connectivity (k) of the co-expression modules, an adjacency matrix (AM) and a Topological overlap matrix (TOM) is obtained. We used the gradient method here and ranged the power values from 1 to 10. When the correlation between *k* and *p*(*k*) reached 0.85, the optimal power value was determined to construct a scale-free topology network. Afterward, a hierarchical clustering dendrogram of the 1-TOM matrix was constructed to stratify the similar gene expressions into different gene co-expression modules. For any modules, since the module Eigengenes (ME) offered the most appropriate interpretation of the gene expression profile, we correlated the ME with clinical features, which included tumor or normal status in this study. Finally, we selected the modules displaying highly positive or negative correlation (according to Moduletrait relationships) as further research goals.

### Interaction with the Modules of Interest and Identification of Prognosis-Related Genes

We extracted the overlapping genes between the interest modules from the TCGAMIBC and GEOMIBC datasets, and then, we presented it as a Venn diagram using the VennDiagram R-package. The TCGAMIBC clinical information was utilized to determine the prognosis-related genes applying the univariate Cox progression analysis. The hazard regression model was utilized to evaluate the prediction performance. The forest plot of prognosis-related genes was constructed by R software.

### Genetic Alteration, Molecular Mechanisms, and Cross-Talk Between Prognosis-Related Hub Genes

We estimated the RNA expression and protein expression level alteration in MIBC comparing normal tissue by using unpaired *t*-test analysis and immunohistochemistry (IHC), respectively. The immunohistochemistry of prognosis-related genes was obtained from the Human Protein Atlas (HPA) database (https://www.proteinatlas.org/). Immunohistochemical data from the same patient ID and antibody types were preferentially selected for comparison. We further downloaded the gene set of the hallmark-related cancer pathways, GO cell components, and molecular functions from the GSEA website (https://www.gsea-msigdb.org/gsea/index.jsp). The TCGAMIBC gene expression profiles were subjected to the Gene Set Variation Analysis (GSVA), a non-parametric, unsupervized method for estimating alteration of gene set enrichment through the samples of an expression data set (Hänzelmann et al., 2013). The Pearson Correlation Coefficient (PCC) was calculated to estimate the correlation between the expression of the prognosis-related genes and the activity of gene sets. Then, the PCC between hallmark-related cancer pathways and prognosis-related genes was visualized by the pheatmap R-package. The relationships (|PCC|>0.6 and *p*-value <0.05) between GO (including molecular function and cell component) gene sets activity and expression of prognosis-related genes were showed by network diagrams constructed by the Cytoscape. Next, we calculated the correlation among the expression of 15 prognosis-related genes by the Hmisc R-package, and then the correlation coefficient diagram was depicted using the corrplot R-package. The protein-protein interaction (PPI) network between 15 prognosis-related genes was established by the STRING website (https://string-db.org/cgi/input.pl) and was visualized using the Cytoscape.

### Consensus Clustering of 15 Prognosis-Related Genes

We collected the RNA-seq expression data of prognosis-related hub genes from the TCGAMIBC dataset to perform unsupervized clustering utilizing the factoextra R-package. The Euclidean distance of different samples was calculated, and then the ward. D2 method was applied to perform the hierarchical clustering. Ultimately, we stratified the TCGAMIBC samples into two clusters and visualized the expression of 15 genes in cluster1/2 using the pheatmap R-package.

### Implementation of Immune Score and Single-Sample Gene Set Enrichment Analysis

We calculated the immunoscore for each patient through the estimate R-package. The fraction of 22 immune cell types for each sample was yielded through cell type identification by estimating relative subsets of RNA transcripts (CIBERSORT). We obtained the gene sets of 24 immune cell types across all tumors from the published literature (Bindea et al., 2013) and then quantified the infiltration levels of these gene sets using the ssGSEA in GSVA R-package. The immune-related gene signature used in this study consisted of activated DCs (aDC), innate immunity, including natural killer (NK) cells, CD56dim NK cells, CD56bright NK cells, dendritic cells (DCs), plasmacytoid dendritic cells (pDC), immature DCs (iDC), neutrophils, mast cells, eosinophils, and macrophages, and adaptive immunity, including B cells, T cells, T central memory cells (Tcm), T effector memory (Tem), CD8 T cells, cytotoxic cells, T follicular helper (TFH), Th1, Th2, Th17, and Treg cells. The ssGSEA score for each immune cell type was standardized by the following equation:

$$\text{score}=\frac{x-\min\left(x\right)}{\max\left(x\right)-\min\left(x\right)}.$$

### Identification of Risk Characteristic Genes

We randomly divided the TCGAMIBC samples into two cohorts, including the training cohort and validation cohort. Meanwhile, characteristic gene signatures were established using the LASSO regression analysis in the TCGA training cohort. The signatures were screened by selecting the optimal penalty parameter l correlated with the minimum 10-fold cross-validation. Then we utilized the coefficients obtained from the LASSO regression algorithm and gene expression value to yield risk score, and the equation was showed as following:

$$\text{Risk}-\text{score}=\text{sum}\left({\text{Coef}}^{*}{\text{Exp}}_{\text{genes}}\right).$$

### Analysis of Gene Set Enrichment Analysis

To validate the correlation between the activity of hallmark pathways and the expression of prognosis-related genes, we separated the TCGAMIBC into two cohorts based on the median expression value of individual prognosis-related gene. Then we conducted the Gene Set Enrichment Analysis in the Hallmark gene set “h.all.v6.2.symbols.gmt” of the MSigDB by using the JAVA program. The algorithm of random sampling was 1,000 permutations. The significant enrichment pathway between the two cohorts was determined by utilizing the false discovery rate of <0.05 and the NES (Normalized Enrichment Score). To explore which hallmark pathways were enriched in cluster1/2 or high/low-risk cohorts, the GSEA was also conducted using the same methods.

### Verification of Survival Prediction

The Kaplan-Meier survival curves were plotted using survival and survminer R-package. The log-rank test was used to estimate the relationship between different objects and patient survival.

### Statistical Analyses

For all analyses, the unpaired *t*-test analysis was used to compare the differences between the two groups, and the log-rank test was used to estimate prognosis differences. Meanwhile, the Pearson method was employed to calculate the correlation coefficient between the two groups. For all analyses, the *p*-value < 0.05 was regarded as a statistically significant difference.

## Results

### Determination of the Most Relevant Module Genes for Muscle-Invasive Bladder Cancer

**FIGURE 1:**
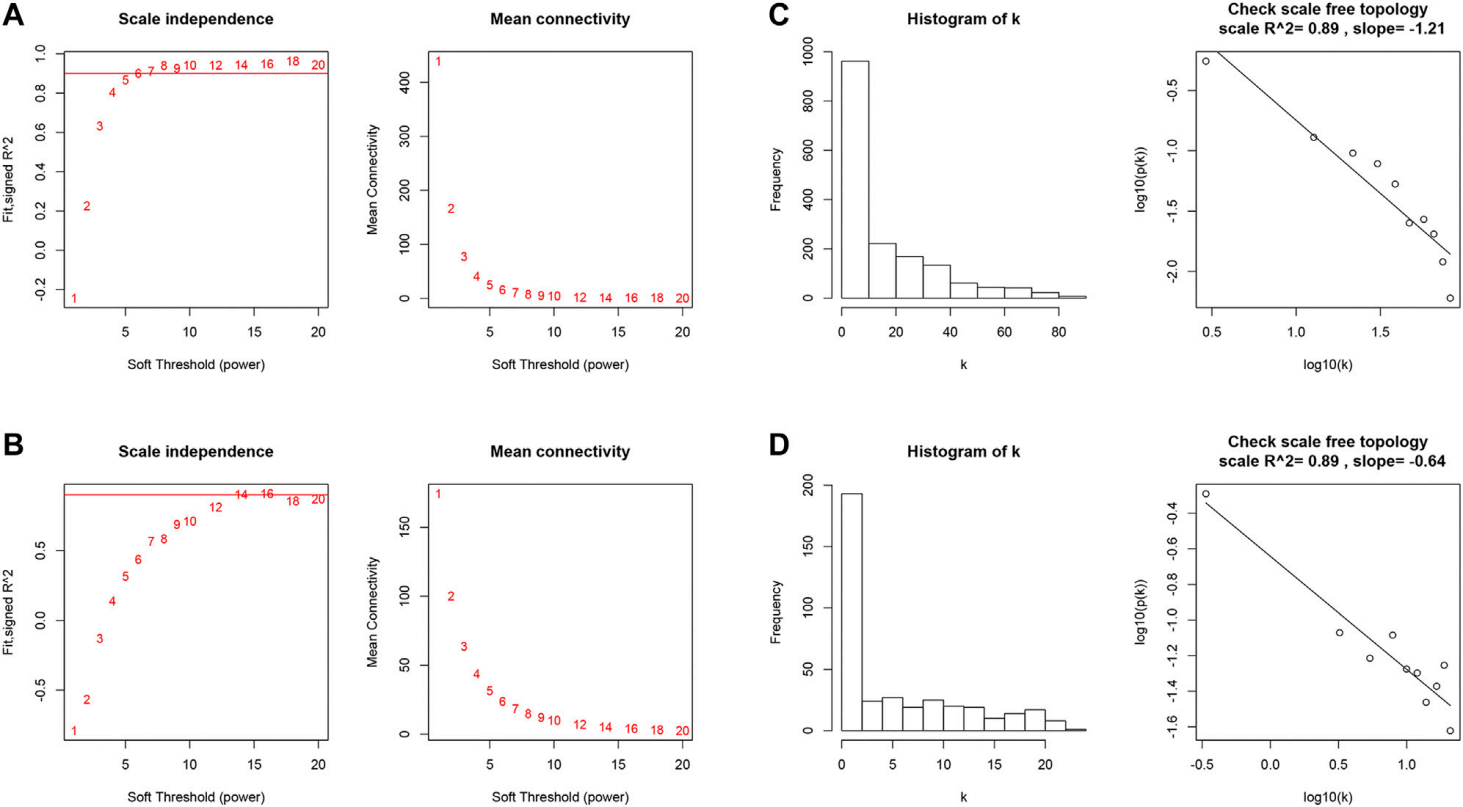
**(A and B)** Analysis of the scale-free fit index and the mean connectivity for various soft-thresholding powers of TCGAMIBC and GSE13507 datasets. **(C and D)** Checking the scale-free topology when β = 6 and 14. K shows the logarithm in the whole network connectivity, *p* **(** *k* **)** represents the logarithm of the corresponding frequency distribution. K is negatively correlated with *p* **(** *k* **)**. The correlation coefficient was 0.89 in both TCGAMIBC and GSE13507 datasets, which represents scale-free topology.

**FIGURE 2:**
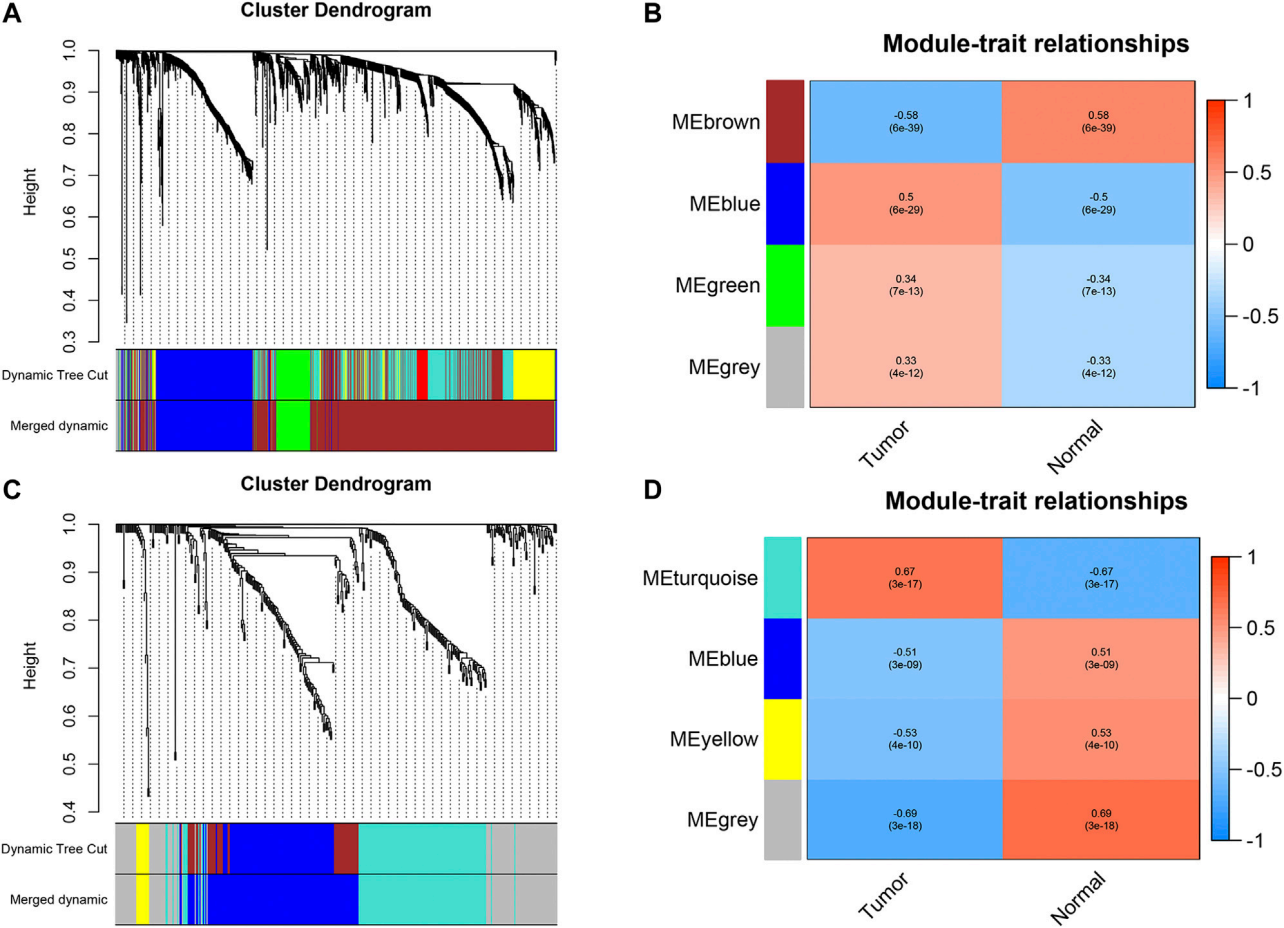
**(A and B)** Dendrogram of all differentially expressed genes in TCGAMIBC and GSE13507 datasets clustered based on a dissimilarity measure (1-TOM). **(C and D)** The heatmaps were depicted to show the correlation between module Eigengenes and clinical status (including tumor and normal status), correlation coefficients and *p*-values were showed in every module.

We first identified 1,664 DEGs and 377 DEGs from the TCGA-MIBC and GSE13507 datasets, respectively. Then we performed a co-expression analysis to construct the co-expression network using WGCNA R-package. In TCGAMIBC and GSE13507 cohorts, a total of four modules were identified via the average linkage hierarchical clustering. To achieve a scale-free co-expression network, we selected the power of β = 6 and 14 in these two cohorts, respectively (Figures 1A,B). We determined the optimal power value and constructed a scale-free topology network when the correlation between *k* and *p*(*k*) reached or exceeded 0.85 (Figures 1C,D). To merge the highly familiar modules, we chose a cut line of <0.25 and a minimum module size of 30 using the dynamic hybrid tree cut method (Figures 2A,B). Next, the interest modules from TCGAMIBC dataset (Brown module: r = −0.58, p = 6*e* − 39; Blue module: r = 0.5, p = 6*e* − 29) and GSE13507 dataset (Turquoize module: r = 0.67, p = 3*e* − 17; Gray module: r = −0.69, p = 3*e* − 18) were found to have the highly correlation (including positive and negative correlation) with the tumor status (Figures 2C,D).

### Identification of Overlapping Genes and Prognosis-Related Genes

**FIGURE 3:**
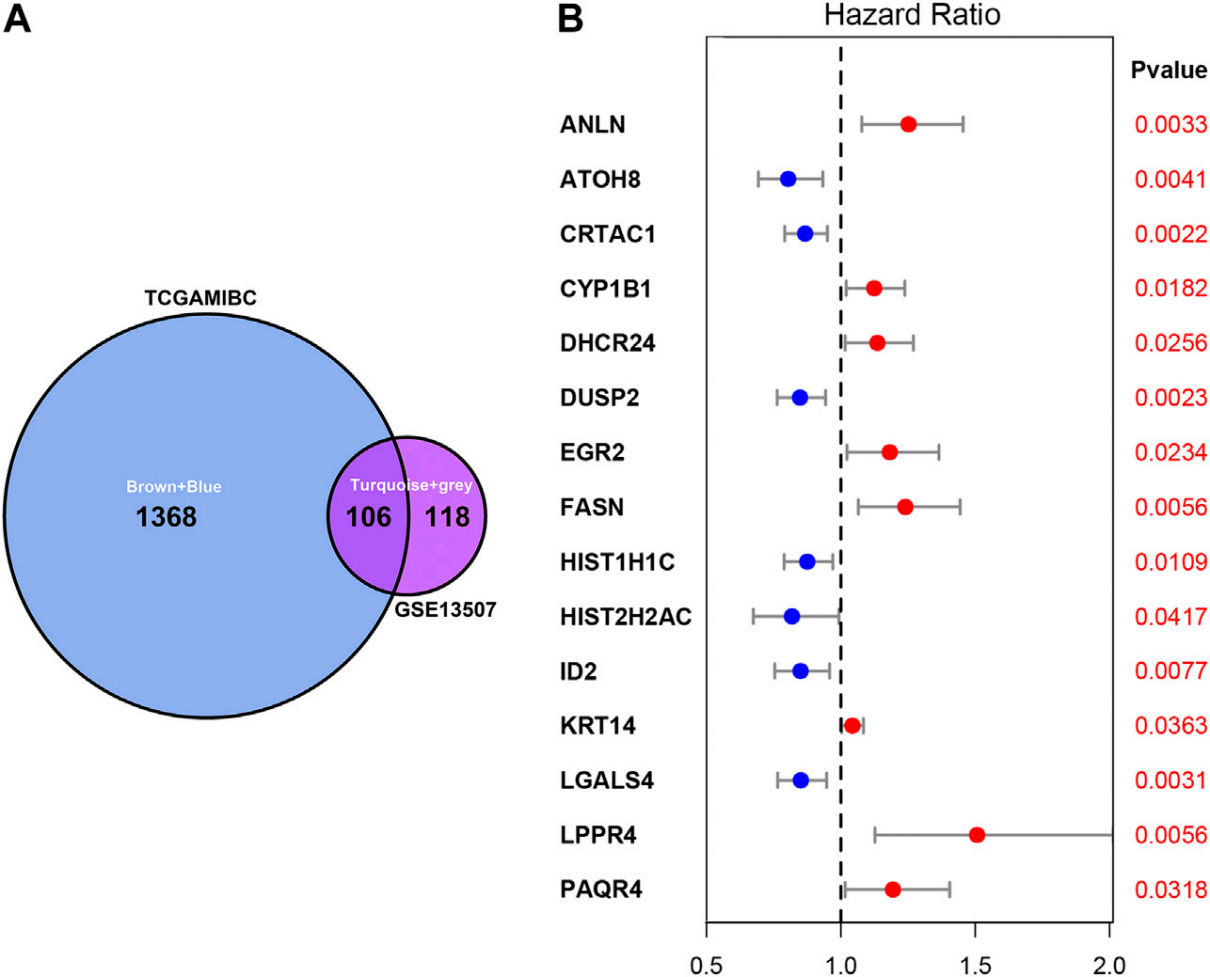
Venn plot of overlapping genes and forest plot of prognosis-related genes in MIBC. **(A)** The intersection of the Venn plot is considered to be co-expressed genes. **(B)** Forest plot shows the distribution of prognosis-related genes hazard ratios in TCGAMIBC. The red point indicates the high hazard ratio, and the blue point indicates the low hazard ratio. *p*-value < 0.05 is regarded as significant and identified by the red font.

**FIGURE 4:**
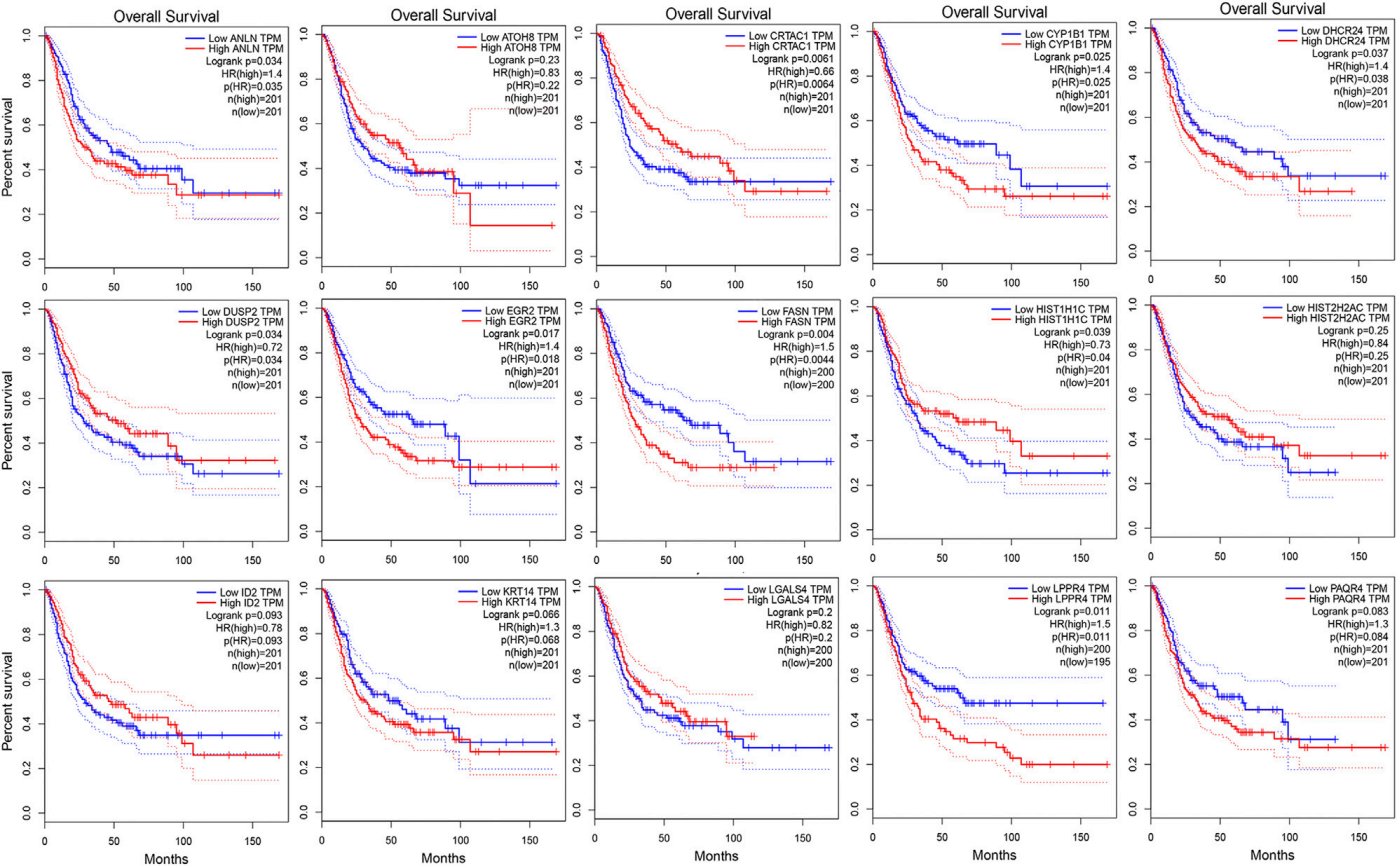
The kaplan-Meier survival curves of patients divided by the expressions of individual prognosis-related genes in MIBC. The *p*-values calculated by the log-rank test are shown.

As shown in Figure 3A, we ultimately identified 1,474 and 224 co-expression genes in the interest module of TCGAMIBC and GSE13507 datasets. A total of 106 overlapping genes were selected for validating the genes of co-expression modules (Figure 3A). To further validate prognosis-related genes, we conducted the univariate Cox regression analysis using the 106 genes and the prognosis information of TCGAMIBC patients. Then 15 genes were finally determined that are closely correlated with the prognosis of MIBC patients (Figure 3B). Among these, we observed some genes were risky prognosis factors (hazard ratio >1, *p*-value < 0.05), including ANLN, CYP1B1, DHCR24, EGR2, FASN, KRT14, LPPR4, and PAQR4. Other genes, including ATOH8, CRTAC1, DUSP2, HIST1H1C, HIST2H2AC, and LGALS4, were shown as protective factors to MIBC patients. We further depicted the Kaplan-Meier plotter using the GEPIA2 database (Figure 4) to investigate the prognostic values of these 15 genes in the MIBC patients. The results suggested that the higher expression level of ANLN, CYP1B1, EGR2, FASN, and LPPR4 were significantly correlated with the worse outcomes of the MIBC patients (*p* < 0.05). In contrast, the lower expression level of CRTAC1, DUSP2, and HIST1H1C was significantly related to the worse outcomes of MIBC patients. However, ATOH8, ID2, KRT14, and LGALS4 showed no significant correlation with the overall survival of MIBC patients.

### Expression Alteration and Cross-Talk of Prognosis-Related Genes

**FIGURE 5:**
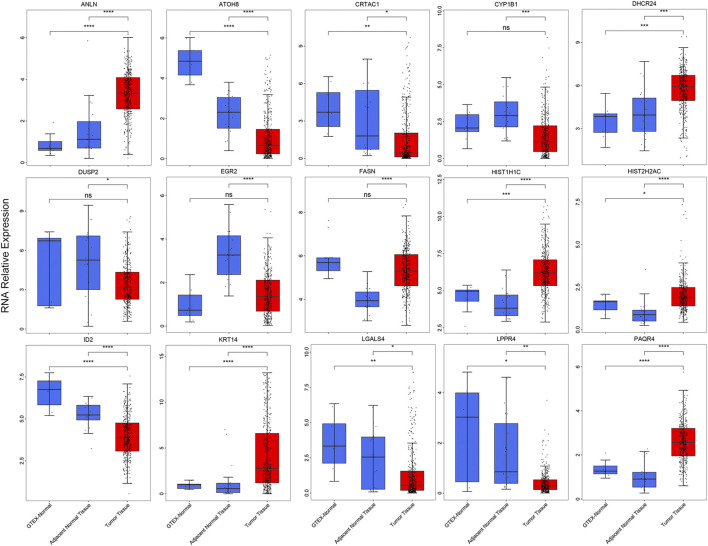
The boxplots show the expression of 15 prognosis-related genes in TCGAMIBC, adjacent non-carcinoma tissues, and GTEx normal tissues, *t*-test was used to calculate the significance level between two groups.

**FIGURE 6:**
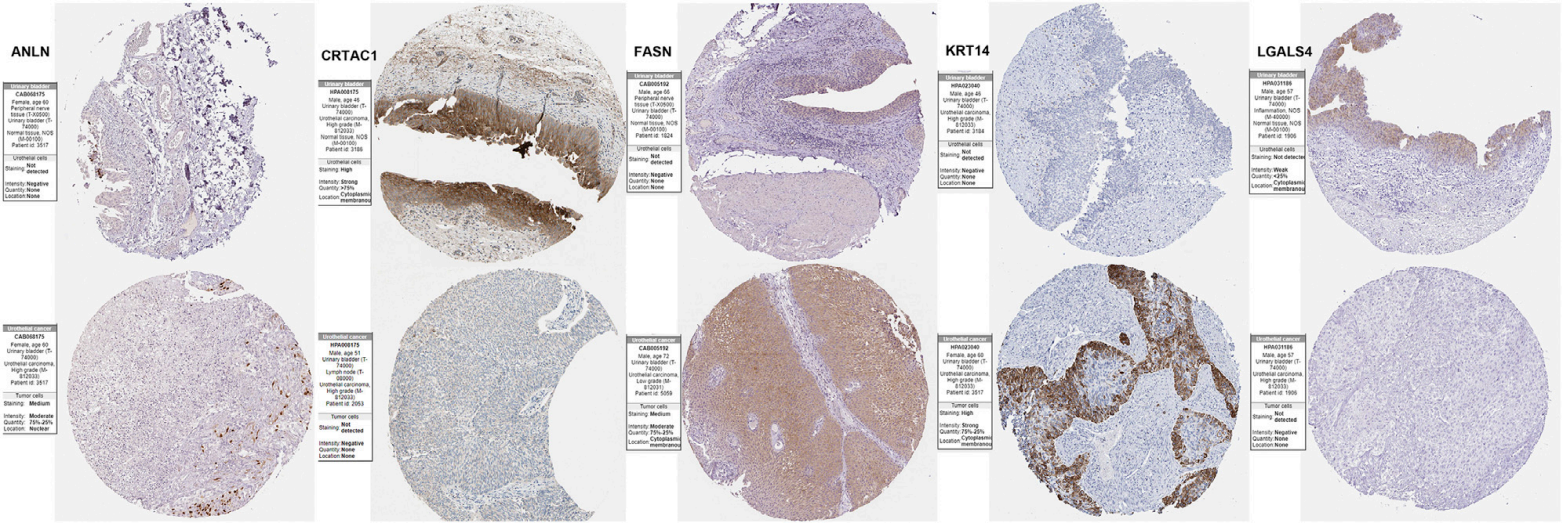
Immunohistochemistry of the prognosis-related genes, including ANLN, CRTAC1, FASN, KRT14, and LGALS4, in MIBC and normal tissues from the human protein atlas (HPA) database.

To validation the RNA-seq expression alteration of 15 prognosis-related genes, we integrated the TCGAMIBC tissue, TCGA adjacent non-carcinoma tissue, and GETX datasets. We found that ANLN, DHCR24, LGALS4, HIST1H1C, HIST2H2AC, KRT14, and PAQR4 in MIBC were consistently up-regulated. Conversely, ATOH8, CRTAC1, ID2, LGALS4, and LPPR4 in MIBC showed consistently down-regulated. However, though the expression of CYP1B1, DUSP2, EGR2, and FASN showed a significant difference between MIBC and adjacent non-carcinoma tissues, but represented no significant difference between MIBC and GTEX normal tissue (Figure 5). Then we further performed the same analysis using the GSE13507 dataset. The results were consistent with that of using the TCGAMIBC dataset (Supplementary Figure S1). Next, we observed the alteration of these gene-encoding protein levels on the HPA database (Figures 6A–E; Supplementary Figure S2). We discovered that the protein level alterations of ANLN, CRTAC1, FASN, KRT14, and LGALS4 were consistent with their RNA-seq expression alteration. However, CYP1B1, DHCR24, HIST1H1C, and HIST2H2AC showed no significant difference, and other gene protein expression data were not identified in the HPA database.

### Cross-Talk Among the Prognosis-Related Genes

**FIGURE 7:**
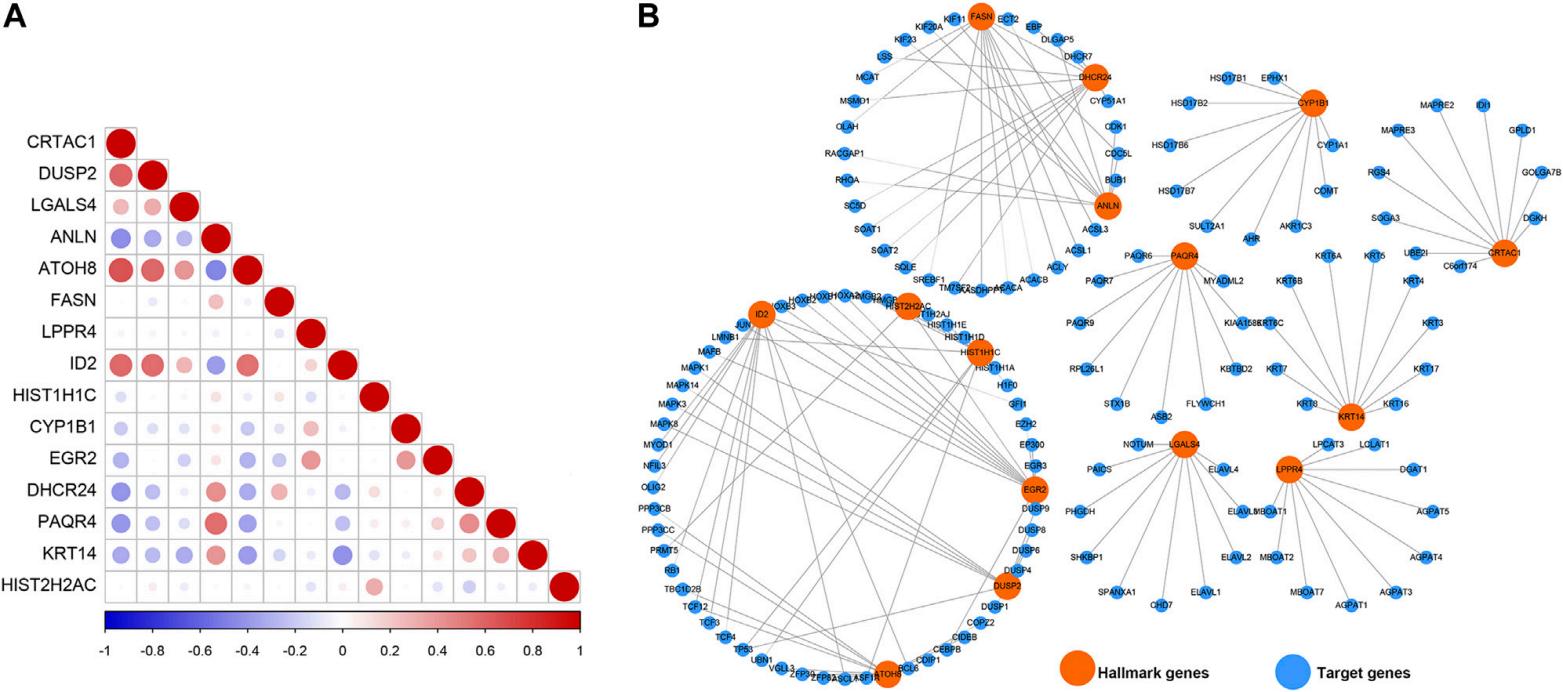
Co-expression relationship and protein-protein interaction network. **(A)** The correlation diagrams show the correlation among 15 prognosis-related genes for MIBC. The positive correlations are colored by red, and negative correlations are colored by blue. The size of the point represents the *p*-value. **(B)** The PPI network diagram shows the proteins that have potential interactions with 15 prognosis-related genes.

To understand the cross-talk between the prognosis-related genes, PCC was calculated to represent the relationship of these genes based on their expression in the TCGAMIBC dataset (Figure 7A). We observed that the expression of CRTAC1 was positively correlated with that of DUSP2, ATOH8, and ID2. ANLN was positively related to PAQR4. DUSP2 was positively correlated to ATOH8 and ID2. However, most of the genes showed no co-expression. Besides, we identified the potential protein that directly interacted with the prognosis-related genes based on the STRING interaction database (Figure 7B). Moreover, we found that FASN interacted with ANLN and DHCR24; HIST2H2AC interacted with HIST1H1C and EGR2; EGR2 interacted with ID2. However, other genes, such as PAQR4, LGALS4, LPPR4, KRT14, CRTAC1, and CYP1B1, showed no interaction with other prognosis-related genes-encoding protein levels.

### Molecular Mechanism of Prognosis-Related Genes

**FIGURE 8:**
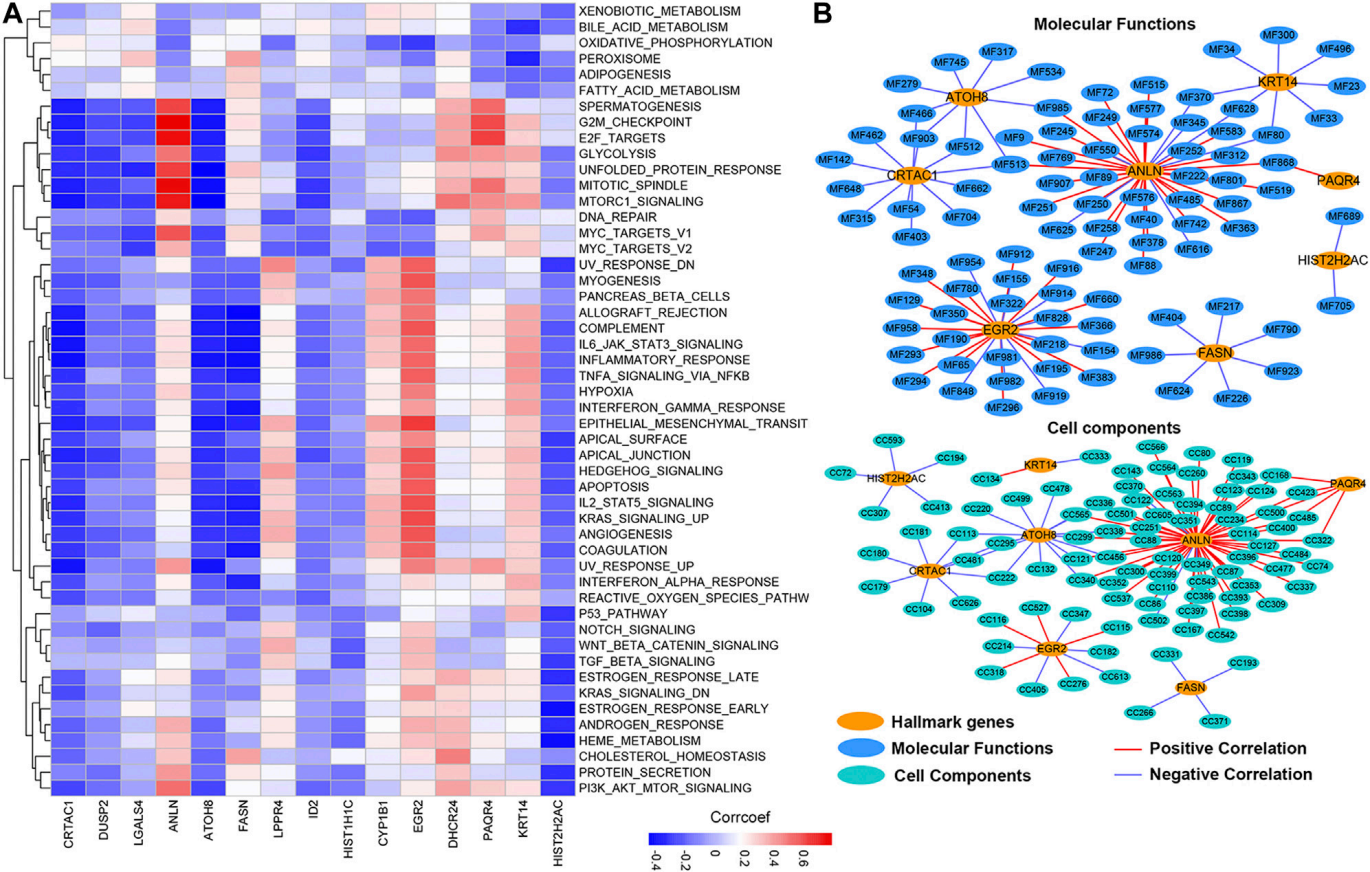
The potential cancer hallmark pathways, molecular functions, and cell components associated with prognosis-related genes. **(A)** The heatmap shows the positive or negative correlations between hallmark-related cancer pathways and genes. Positive correlations are shown by red; negative correlations are shown by blue. **(B)** The PPI network diagram shows the cell components and molecular functions that have potentially regulation by the prognosis-related genes.

To further explore the molecular mechanisms by which prognosis-related genes are responsible for cancers, we calculated the correlation between the expression of prognosis-related genes and the activity score of 50 hallmark-related cancer pathways. We found that different genes were correlated with varying pathways of cancer (Figure 8A; Supplementary Table S1). The expression of CRTAC1, DUSP2, ATOH8, and HIST2H2AC showed a significantly negative correlation with the activity of most hallmark-cancer pathways. Interestingly, the expression of ANLN and PAQR4 was highly correlated with the activity of G2M checkpoint, E2F targets, mitotic spindle, Mtorc1 signaling, and MYC target V1 pathways. We conducted the same correlation between prognosis-related genes and molecular function (MF) and cell component (CC). The networks were depicted to show the correlations using the Cytoscape software (Figure 8B; PCC >0.60, PCC < -0.4, and *p*-value < 0.05). We found only eight of prognosis-related genes were highly correlated with the activity of MIBC multiple MFs and CCs (Detail in Supplementary Table S2). Among these, we observed that more MF and CC were highly correlated with ANLN, suggesting that ANLN may play a pivotal role in regulating MIBC development. To further validate the potential regulatory mechanisms of ANLN, resulting in MIBC, we performed gene set enrichment analysis (GSEA). On the basis of the expression of ANLN, we stratified the data into two groups (including high ANLN expression and low ANLN expression cohort) and subjected the data to the GSEA program based on the JAVA. The results showed that G2M checkpoint, E2F targets, mitotic spindle, Mtorc1 signaling, and MYC target V1 were correlated with high ANLN expression cohort (Supplementary Figure S3), which were consistent with the result of Figure 8A, suggesting that the P13K/AKT/mTOR/MYC/E2F/G2M pathways might be main mechanisms for ANLN to regulated the development of MIBC.

### Consensus Clustering for Prognosis-Related Genes and Distinct Immune Cell Infiltration Level of Different Clusters

**FIGURE 9:**
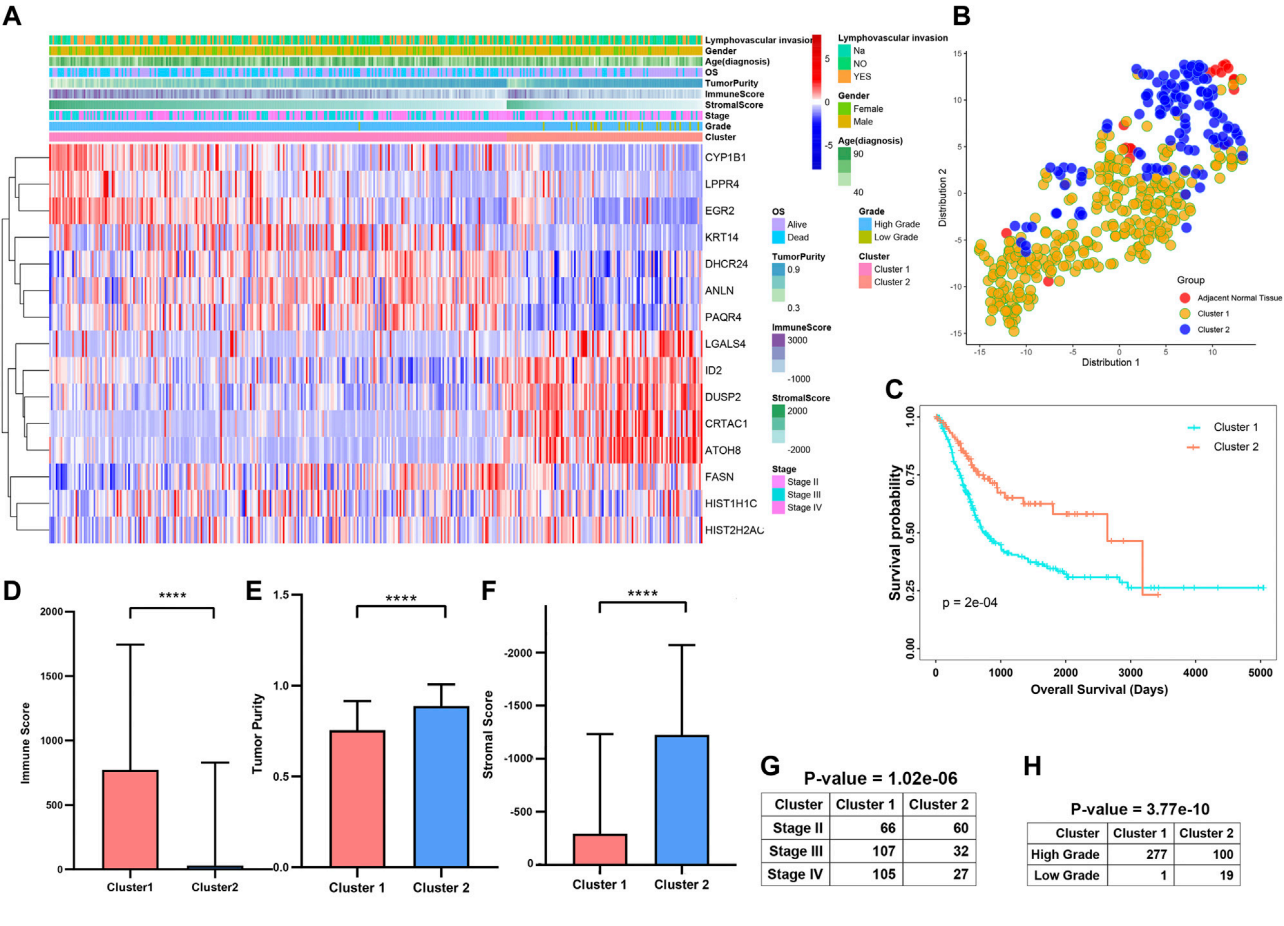
Differential clinicopathological features and survival of patients in Cluster1/2 Subtypes in TCGAMIBC Cohort. **(A)** Heatmap and clinicopathologic features of the two clusters (cluster1/2). **(B)** The t-SNE analysis supported the stratification into two MIBC clusters. **(C–G)** The comparison of the immune score, tumor purity, stromal score, stage, and grade in cluster1/2. **(H)** Kaplan-Meier survival curves of patients in MIBC cluster1 and cluster2. The *p*-values calculated by the log-rank test are shown. *p < 0.05, **p < 0.01, ***p < 0.001, ****p < 0.0001.

The progression and prognosis of cancers are usually regulated with multiple genes. After that, we conducted a consensus clustering based on the expression of 15 prognosis-related genes (Figure 9A). A total of 402 patients with MIBC were separated into two subtypes, namely cluster1 (n = 278) and cluster2 (n = 119). We found the expression of CYP1B1, LPPR4, ANLN, PAQR4, KRT14, and DHCR24 were higher in the cluster1 than that in the cluster2. To validate the subclasses’ assignments, we further performed t-SNE to decrease the dimension of features and found the cluster designations were largely concordant with different-dimensional t-SNE distribution patterns (Figure 9B). Moreover, we found that the prognosis of MIBC patients in cluster1 was worse than that in cluster2 (Figure 9C, *p*-value < 0.0001). To investigate the effect of prognosis-related genes on the tumor immune microenvironment (TIME) of MIBC, we evaluated the immune score, immune infiltration level, and the fraction of 22 immune cells between the cluster1 and cluster2. We found the two clusters represented a significant difference in immune score, purity of tumors, and stromal scores (Figures 9D–F). Moreover, the immune infiltration level of most immune cells in cluster1 was higher than that of cluster2 (Supplementary Figure S4A). Besides, we observed the scale of a fraction of B cell naive, CD4 memory T cell, NK cells activated, Macrophage M0/M1/M2, and Neutrophils in cluster1 were significantly higher than that of cluster1 (Supplementary Figure S4B). However, the fraction scale of Dendritic cells resting/activated, B memory cells, Regulatory T cells, and CD4 naive T cells in cluster1 were lower than that in cluster2. Furthermore, the proportion of patients with higher stages and higher grades was significantly higher in Cluster1 than that in Cluster2 (Figures 9G,H).

### Construction and Immune Characteristic of Risk Signatures

**FIGURE 10:**
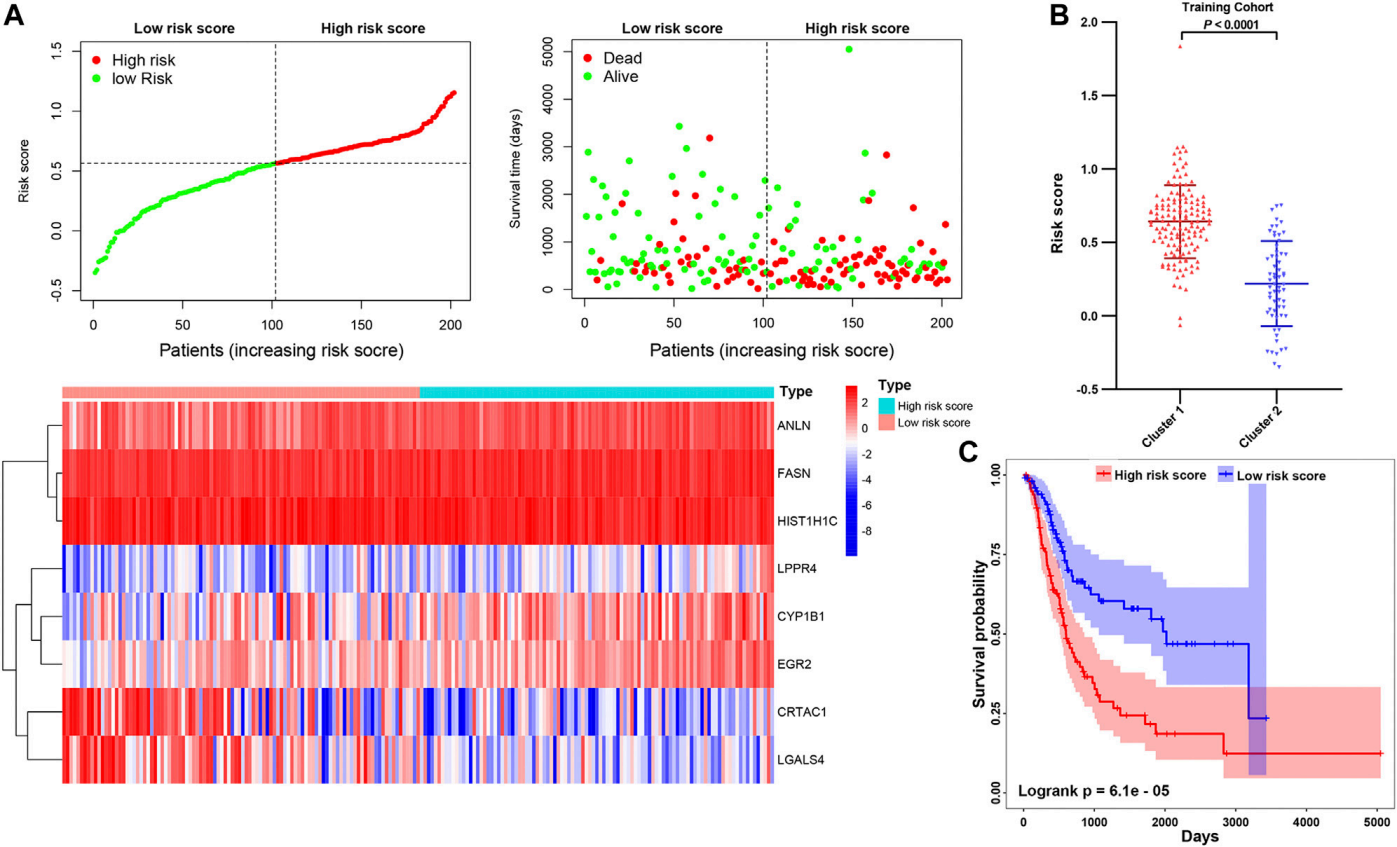
**(A)** Distribution of risk score, OS, and OS status and heatmap of the eight prognostic gene signatures in the TCGA training cohort. **(B)** The comparison of the risk score in cluster1/2. **(C)** Kaplan-Meier survival curves of MIBC patients in high-/low-risk cohorts. The *p*-values calculated by the log-rank test are shown.

To further explore the contribution of these prognosis-related genes to MIBC patients. We divided the 400 MIBC patients into the TCGA training cohort (200 patients) and the validation cohort (200 patients) at a 5:5 ratio. The baseline characteristics among the TCGA training and validation cohorts, including age, gender, T stage, N stage, M stage, grade, TNM stage, and cluster stratified above, were not statistically different (Supplementary Figures S5A,B; all *p* > 0.05). Furthermore, the T-SNE distribution patterns based on the expression of prognosis-related genes in the training and the validation cohort also were not different (Supplementary Figure S5C). To predict the clinical outcome of prognosis-related genes in MIBC patients precisely, we performed the least absolute shrinkage and selection operator (LASSO) regression analysis based on the expression values of 15 prognosis-related genes in the TCGA training cohort. Eight characteristic genes, namely ANLN, FASN, HIST1H1C, LPPR4, CYP1B1, EGR2, CRTAC1, and LGALS4, were identified (Supplementary Figures S6A,B). The risk scores of the MIBC training and validation cohort were determined using the coefficients obtained by the LASSO algorithm, and the equation is as: −0.064 * CRTAC10.071 * LGALS4 + 0.028 * ANLN +0.11 * FASN + 0.22 * LPPR4 − 0.045 * HIST1H1C + 0.018 * CYP1B1 + 0.098 * EGR2. Afterward, patients were divided into high- and low-risk cohorts based on the median risk score. The distribution of the risk scores, OS, OS status, and expression profiles of the eight characteristic-genes-based signatures in TCGA training and validation cohorts is displayed (Figure 10A; Supplementary Figure S7A). The heatmap results indicated that risky genes, including ANLN, FASN, LPPR4, CYP1B1, and EGR2, were highly expressed in the high-risk cohort. In contrast, the expression levels of protective genes, including CRTAC1, LGALS4, and HIST1H1C, were upregulated in the low-risk cohort. Meanwhile, we found that the risk score in cluster1 with a worse outcome is higher than cluster2 (Figure 10B; Supplementary Figure S7B). The OS of patients in the low-risk cohort was longer than that of the high-risk cohort (*p* < 0.0001, Figure 10C; Supplementary Figure S7C). To assess the prognostic accuracy of the eight identified risk signatures, we performed 3- and 5-years receiver operating characteristic (ROC) curve analyses by comparing the respective AUC values. In the TCGA training cohort, the 3- and 5-years AUC values for the eight risk signatures were 0.705 and 0.722, respectively (Supplementary Figure S8A). In the TCGA validation cohort, the 3- and 5-years AUC values for the eight risk signatures were 0.708 and 0.725, respectively (Supplementary Figure S8B). The AUC values showed that the signatures of eight characteristic-genes had a favorable discrimination performance for the prognosis of patients with MIBC.

### Relationships Between Risk Score, Immune Infiltration and Clinical Information

**FIGURE 11:**
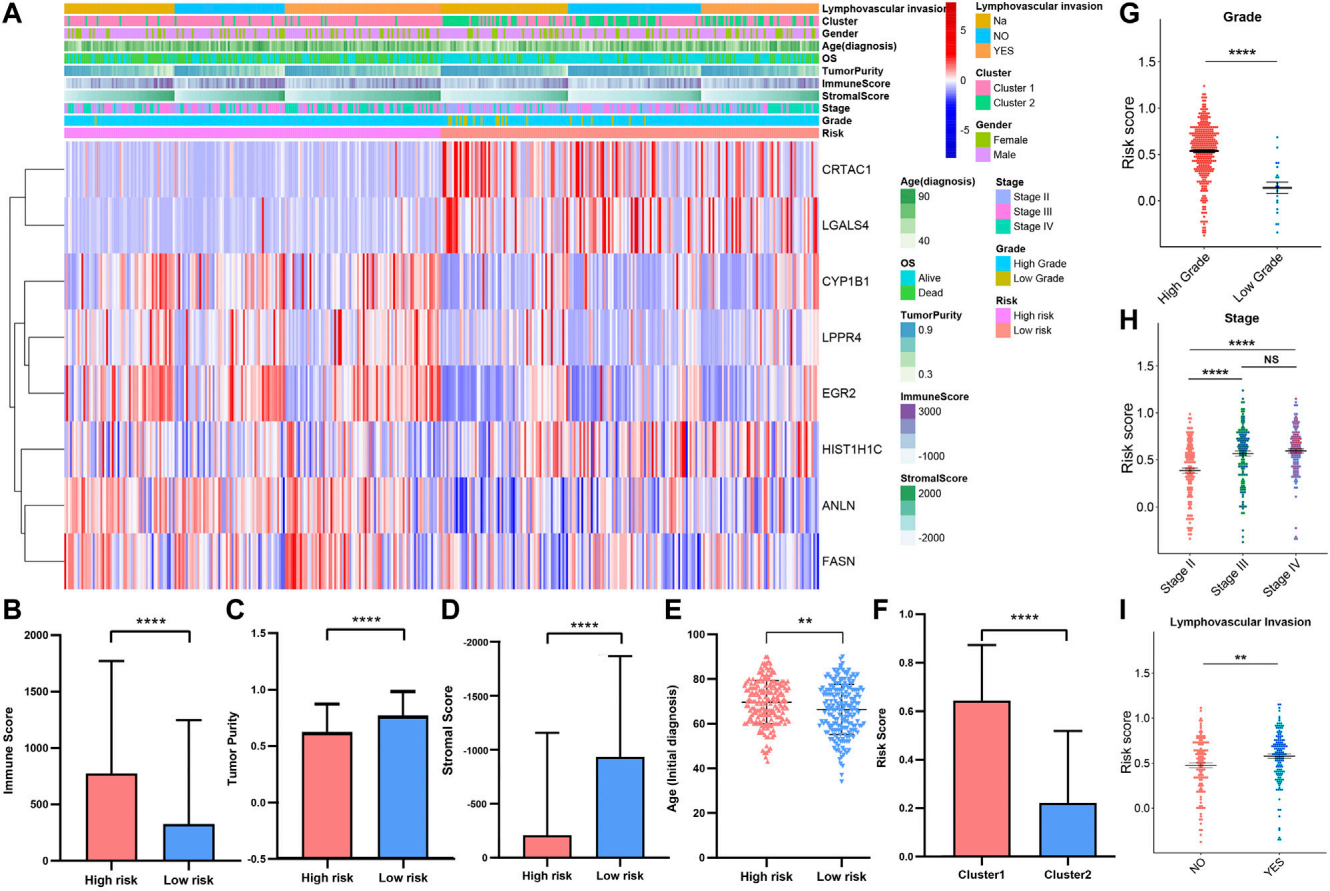
**(A)** Heatmap and clinicopathologic features of high- and low-risk groups. The comparison of immune score **(B)**, tumor purity **(C)**, stromal score **(D)**, age **(E)** in high-/low-risk cohorts. The difference of risk score in various cluster **(F)**, grade **(G)**, stage **(H)**, lymphovascular invasion **(I)**. *p < 0.05, **p < 0.01, ***p < 0.001, ****p < 0.0001.

To investigate the correlation between risk score, immune infiltration levels, and clinical information. We integrated the TCGAMIBC data of the training cohort and validation cohort (Figure 11A). The expression of risky genes, such as ANLN, FASN, CYP1B1, EGR2, and LPPR4 in high-risk-cohort, is higher than that of low-risk-cohort. Conversely, the expression of protective genes, such as HIST1H1C, CRTAC1, and LGALS4 in low-risk-cohort, is higher than that in high-risk-cohort. These results were consistent with Figure 10A and Supplementary Figure S7A. Meanwhile, we found the differences in terms of immunoscore (Figure 11B; *p* < 0.0001), purity of tumor (Figure 11C; *p* < 0.0001), stromal score (Figure 11D; *p* < 0.0001), and ages (Figure 11E; *p* < 0.01) between the high- and low-risk cohorts was significant. Moreover, the risk score in cluster1 was distinctly higher than that in cluster2 (Figure 7F; *p* < 0.0001), and the risk score increased along with the histological grade and stage increased (Figures 7G,H; *p* < 0.05). In MIBC patients with the lymphovascular-invasion, the risk score was distinctly higher than that of no-lymphovascular-invasion (Figure 7I; *p* < 0.0001). Furthermore, the infiltration level of most immune cells in high-risk-cohort was higher than that of low-risk-cohort (Supplementary Figure S9A). The fraction scale of 22 immune cells in the two risk cohorts was basically consistent with that in two clusters (Supplementary Figure S9B). These findings revealed that the risk score was significantly associated with cluster1/2, grade, stage, age, lymphovascular-invasion, and immune infiltration levels in MIBC patients.

### Immune Checkpoint Alterations and Immunoregulation Mechanisms

**FIGURE 12:**
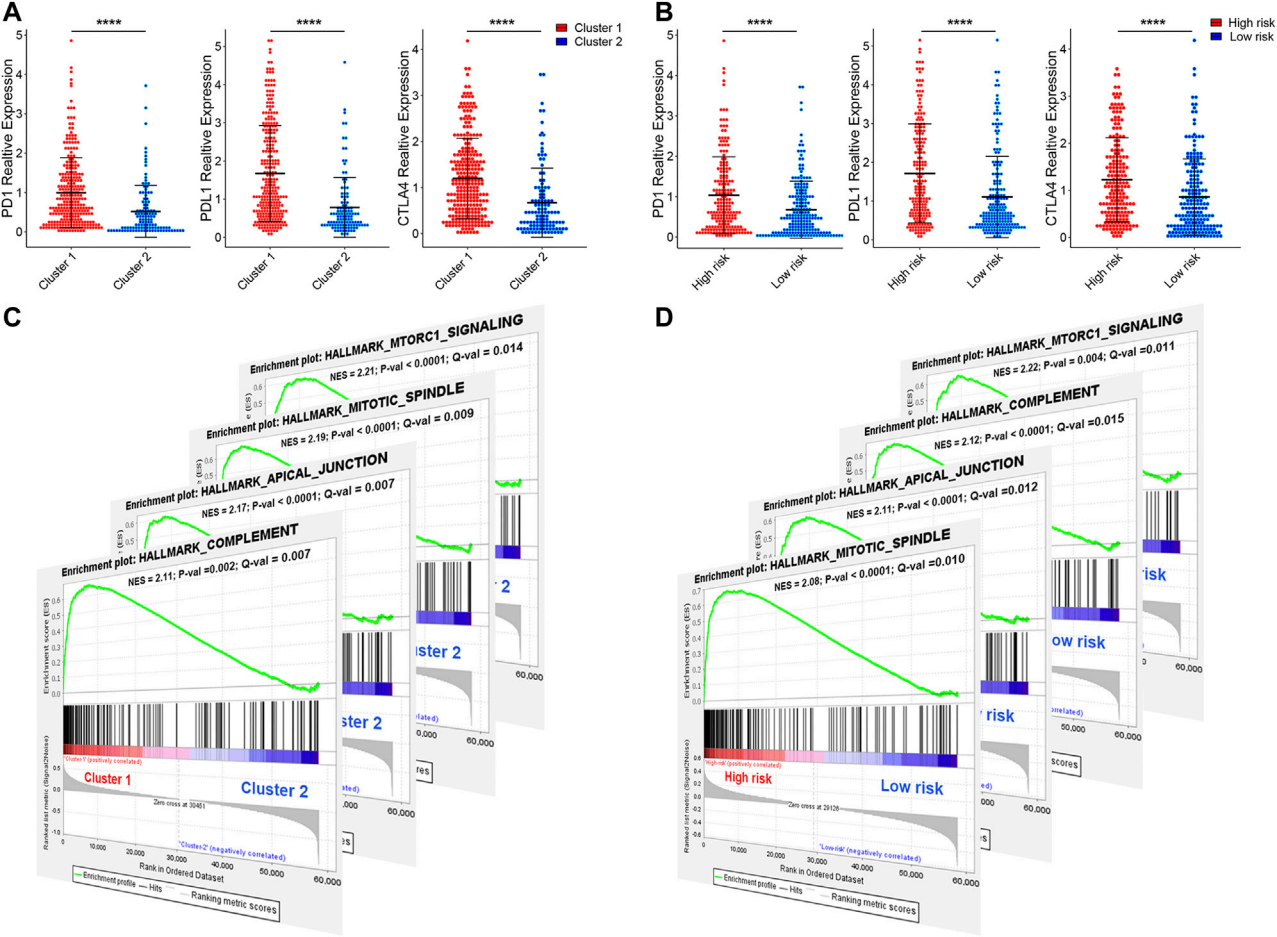
The expression of immune checkpoint and gene set enrichment analysis. **(A)** The expression of PD1, PDL1, and CTILA4 in cluster1/2 and high-/low-risk cohorts. **(B)** The GSEA showed that G2M checkpoint, mTORC1 signaling, and PI3K/AKT/mTOR signaling are differentially enriched in cluster1 and high-risk-cohort. NES, Normalized Enrichment Score; NOM p-val, Normalized *p*-value; FDR q-val, False Discovery Rate q-value. *p < 0.05, **p < 0.01, ***p < 0.001, ****p < 0.0001.

Programmed cell death 1 (PD-1, also known as CD279), programmed cell death 1 ligand 1 (PD-L1, also known as CD274), and CTIL-4, are common immune checkpoints (Sun et al., 2018; Dermani et al., 2019; Qin et al., 2019), have been described in bladder cancer (Bellmunt et al., 2017; Le Goux et al., 2017; Rouanne et al., 2018). To explore the expression of PD-1, PD-L1, and CTIL-4 in cluster1/2 and high-/low-risk-cohort, we conducted unpaired *t*-test analysis (Figures 12A,B). Interestingly, we found that the expression of PD-1, PD-L1, and CTIL-4 in cluster1 and high-risk-cohort were higher than that of cluster2 and low-risk-cohort (all *p* < 0.0001), revealing subtype analysis and the construction of risk-cohort could be a good stratification method to MIBC patients whether to conduct immunotherapy. To elucidate the potential regulatory mechanisms resulting in differences in the TIME and immune-checkpoint in the two clusters and the two risk cohorts, we conducted differential expression analysis comparing cluster1 or high-risk-cohort with cluster2 or low-risk-cohort, respectively (Supplementary Figure S10A). We ultimately screened cluster1-related DEGs and high-risk-related DEGs. Interestingly, we found the cluster1-related DEGs contained almost all high-risk-related DEGs (Supplementary Figure S10B), and the log2 fold change of these overlapping genes between these two groups was significantly consistent (Supplementary Figure S10C and Table S3), suggesting that patients in cluster1 or high-risk-cohort possess uniform the characterization of genetic alteration. Finally, we performed GSEA based on cluster1/2 and high-/low-risk-cohort. The results showed that the consistent malignant hallmark pathways of tumors, including Mitotic spindle, mTORC1 signaling, Complement signaling, and Apical junction pathway, were dynamically correlated with the cluster1 and high-risk-cohort (Figures 12C,D). Hence, a strong association could be considered that the Mitotic/mTORC1/Complement/Apical junction signaling pathways might be implicated in the distinct TIME of cluster1/2 and high/low-risk-cohort.

## Discussion

In this study, we first screened 106 overlapping genes with significant expression alteration in both the TCGAMIBC and GSE13507 databases using integrated bioinformatics analysis. Then we conducted the univariate Cox progression analysis to identified 15 prognosis-related genes, including ANLN, CYP1B1, DHCR24, EGR2, FASN, KRT14, LPPR4, PAQR4, ATOH8, CRTAC1, DUSP2, HIST1H1C, HIST2H2AC, and LGALS4. ANLN, CYP1B1, DHCR24, EGR2, FASN, KRT14, LPPR4, and PAQR4 showed as risky factors in MIBC. However, ATOH8, CRTAC1, DUSP2, HIST1H1C, HIST2H2AC, and LGALS4 showed as protective factors. Moreover, the expression level of these genes could predict different prognosis of MIBC patients. Then we calculated the correlation between the expression value of prognosis-related genes and the activity score of 50 hallmark-related pathways, molecular functions, and cell components. These prognosis-related genes showed heterogeneous biological functions or molecular mechanisms, indicated that MIBC development is subjected to multifaceted regulation. Interestingly, we found that ANLN showed a stronger correlation with multiple cancer pathways, biological functions, and cell components, suggesting that ANLN may be a pivotal gene in MIBC development, consistent with previous findings (Zeng et al., 2017; Wu et al., 2019). In addition, PAQR4, HIST1H1C, and HIST2H2AC seem to play a specific role in MIBC, but they still need to validation further, because no report about these genes contributes to MIBC. The progression and prognosis of cancers are usually regulated with multiple genes (Chan et al., 2008; Shimoni, 2018). We further identified two subtypes of MIBC, that is, cluster1 and cluster2, by conducting a consensus clustering based on the expression of 15 prognosis-related genes. The MIBC patients in cluster1 or 2 represented distinct outcomes and different clinicopathological features, immunoscore, stage, grade, and immune cell infiltration levels. We also yielded eight prognostic risk signatures from the 15 prognosis-related genes, which effectively stratified the TCGAMIBC patients into high- and low-risk cohorts. The high- and low-risk cohorts were also related to distinct prognosis, clustering subtypes, immunoscore, stage, age, and grade. These results revealed that both the consensus clustering and the constitution of risk signatures on the basis of the expression of 15 prognosis-related genes are feasible stratification methods for MIBC patients. Notably, among these risk signatures, ANLN was identified as a promising prognostic biomarker that could be used to stratify different risks of BLCA (Zeng et al., 2017). FASN catalyzing the terminal steps in the *de novo* biogenesis of fatty acids is correlated with low survival and high disease recurrence in patients with bladder cancer (Tao et al., 2019). Common CYP1B1 variants acted as risk factors for bladder cancer, which increases with occupational exposure (Salinas-Sánchez et al., 2012). LGALS4 could predict a good prognosis of urothelial carcinoma of the bladder and restrained the growth and migration of urothelial carcinoma of the bladder cells (Ding et al., 2019). These findings revealed that the dysregulation of these four risk genes served as pivotal functions in bladder cancer (including NMIBC and MMIBC). However, the dysregulation of the other risk genes in MIBC remains ambiguous, which needs to validate further. Moreover, we observed the immune infiltration levels of most 24 immune cells in the cluster1 and high-risk-cohort were higher than that in cluster2 and low-risk-cohort. Interestingly, the patient prognoses in cluster1 and high-risk-cohort with an activated immune system were worse than that in cluster2 and high-risk-cohort. Indeed, in urothelial carcinoma, tumors are highly infiltrated by Treg cells (Loskog et al., 2007), which often correlates with a poor prognosis (Tsai et al., 2014). Previous results from several *in vitro* and *in vivo* studies showed that high infiltration of macrophages with an unfavorable M2 profile was associated with a poor outcome in patients with NMIBC and MIBC (Ajili et al., 2013; Takeuchi et al., 2016). High infiltration levels of Treg cells, TH2 CD4^+^ T cells, MDSCs, M2 macrophages, and neutrophils are often associated with poor prognosis (Becht et al., 2016; Fridman et al., 2017). In addition, at a steady-state, the ability of the immune system to maintain equilibrium between amplifying and restraining the immune response is essential to avoid potential autoimmunity and tissue damage while generating a successful immune defense (Schneider et al., 2019). When the immune system becomes activated, the upregulation of inhibitory receptors is a necessary feedback mechanism to avoid pathogenic inflammatory responses and autoimmune disorders (Zhang and Vignali, 2016). Hence, the activated immune system of MIBC patients in cluster1 and high-risk-cohort generated an adverse effect on the prognosis of MIBC patients, which may be involved in the imbalance between the activated and suppressed immune responses. In the multifaceted immune regulation of bladder cancer, PD-1, PD-L1, and CTLA4 are the primary immune checkpoints. In our results, the expression of PD-1, PD-L1, and CTLA4 in cluster1 and high-risk-cohort were higher than those in cluster2 and low-risk-cohort. Three studies found that PD-L1 expression increased with tumor stage and grade and was associated with worse overall survival, both in NMIBC and MIBC (Inman et al., 2007; Nakanishi et al., 2007; Huang et al., 2015). In a large cohort, including patients with NMIBC or MIBC, a high level of PD-L1 expression on tumour-infiltrating immune cells was an independent predictor of reduced overall survival and RFS (Wang et al., 2019). In patients with MIBC, a high level of CD8+PD-1high T cells in urine was associated with reduced RFS (Wong et al., 2018). Compared with anti-PD-1 (nivolumab) treatment alone, the combination of anti-PD-1 and anti-CTLA4 (ipilimumab) treatment resulted in an ORR of 38% (vs. 25.6%), a median tumor lesion change from baseline of −30.0% (vs. +1.9%), and median overall survival of 15.3 months (vs. 9.9 months), at the best dose combination (Sharma et al., 2019). These findings indicated that immune therapy against these immune checkpoints is an important clinical treatment strategy. Hence, we revealed that the patients in the cluster1 and high-risk cohort might be sensitive to the treatment of these immune checkpoints. For these patients, anti-PD-1, anti-PD-L1, and anti-CTLA4 may be able to achieve better clinical expectations. Finally, the results of the GSEA showed that the malignant hallmarks of tumors, including Mitotic spindle, mTORC1 signaling, Complement signaling, and Apical junction pathway, were dynamically correlated with the cluster1 and high-risk-cohort. The relationships between these hallmark-related cancer pathways and immune regulation have previously been revealed (Wang et al., 2016; Zhao et al., 2019). Hence, the Mitotic/mTORC1/Complement/Apical-junction signaling pathways might be implicated in the different TIME of MIBC cluster1/2 and high/low-risk-cohort.

In summary, we integrating WGCNA with differential gene expression analysis identified 106 MIBC signature genes and further identified 15 MIBC prognosis-related genes. We systematically analyzed the genetic alterations, molecular mechanisms, and clinical relevance of these 15 identified genes. We found these 15 genes showed different genetic alterations, involved in various molecular mechanisms, and were correlated with prognosis and recurrence of clinical cancer patients. We further did consensus clustering and risk modeling of 15 prognosis-related genes and shown that the prognosis, immune infiltration status, stage, and grade of MIBC patients were significantly different in cluster1/2 and high/low-risk-cohort. The expression of PD-1, PD-L1, and CTLA4 was significantly up-regulated in cluster1/high-risk-cohort than that in cluster2/low-risk-cohort. These results revealed that a feasibility stratification methods to MIBC patients based on these 15 prognosis-related gene expressions and provides a possibility to screen out MIBC patients who are sensitive to immunotherapy and help for the future treatment strategies of these patients. However, our researches also had limitations about the classification of MIBC patients. Although we provided a comprehensive bioinformatics analysis to identify potential prognosis-related genes to MIBC patients and conduct significant stratification, it may not be very accurate for each patient with MIBC. Moreover, the molecular mechanisms for the prognosis-related genes that affected the immune infiltration levels and prognosis of MIBC patients should be further validated through many clinical trials.

## Data Availability Statement

The original contributions presented in the study are included in the article/Supplementary Material, further inquiries can be directed to the corresponding authors.

## Author Contributions

ZZ and ED: conceptualization and methodology. JL and YL: software and data curation. JL and SL: validation. JL, SL, and FS analyzed the data. JL and YL: writing-original manuscript preparation. ZZ and ED: review, editing, and supervision.

## Funding

This work was supported by The National Natural Science Foundation of China (NO. 21577097).

## Conflict of Interest

The authors declare that the research was conducted in the absence of any commercial or financial relationships that could be construed as a potential conflict of interest.
