# Supplementary material for: Identification and Immunocorrelation of Prognosis-Related Genes Associated With Development of Muscle-Invasive Bladder Cancer
Source: Front Mol Biosci. 2021 Jan 29;7:598599. doi: 10.3389/fmolb.2020.598599 (PMC7884823; doi:10.3389/fmolb.2020.598599)
Supplement: Supplementary file 1 [file image1.pdf]

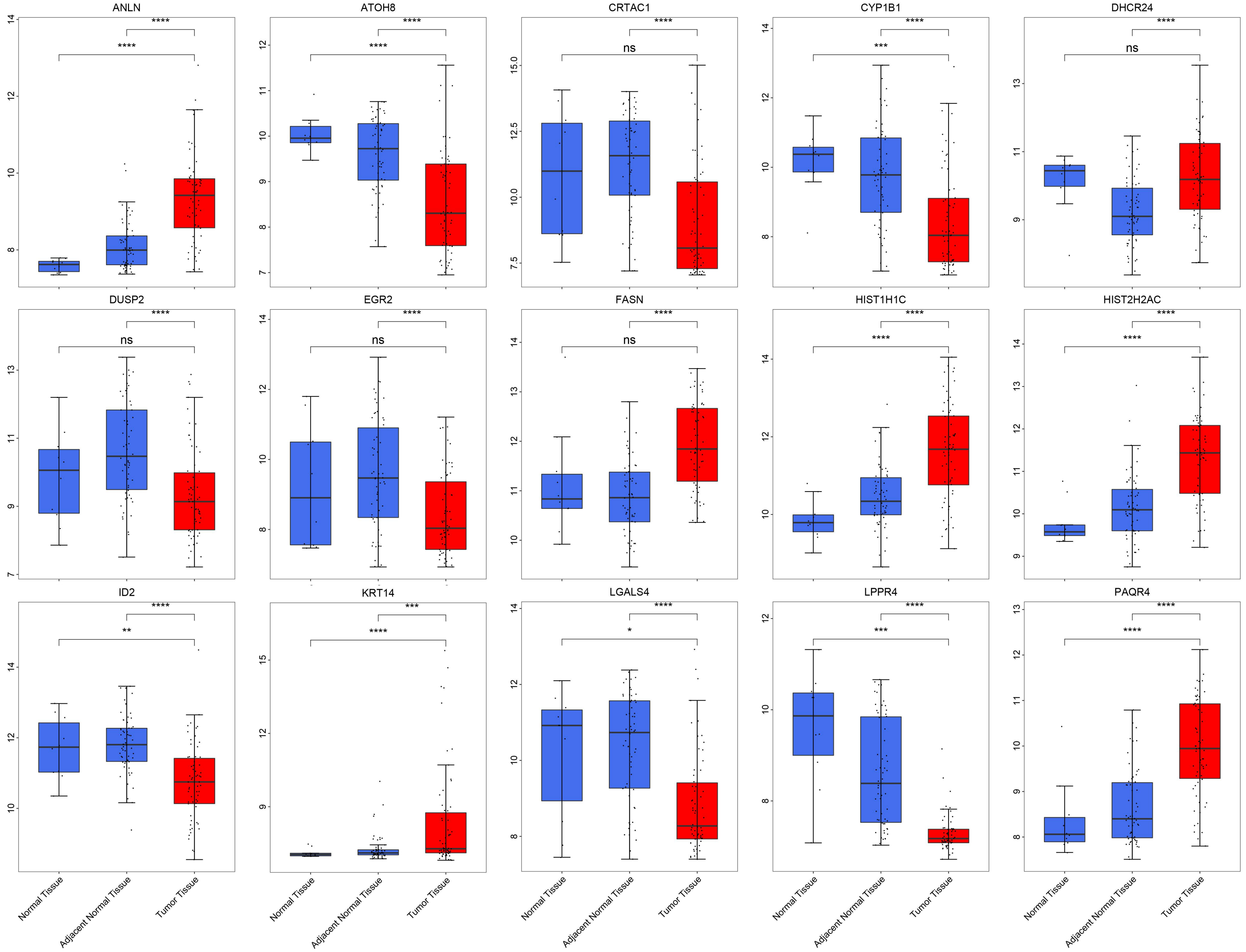
ANLN
ATOH8
CRTAC1
CYP1B1
DHCR24
DUSP2
EGR2
FASN
HIST1H1C
HIST2H2AC
ID2
KRT14
LGALS4
LPPR4
PAQR4
Normal Tissue
Adjacent Normal Tissue
Tumor Tissue

FIGURE S1

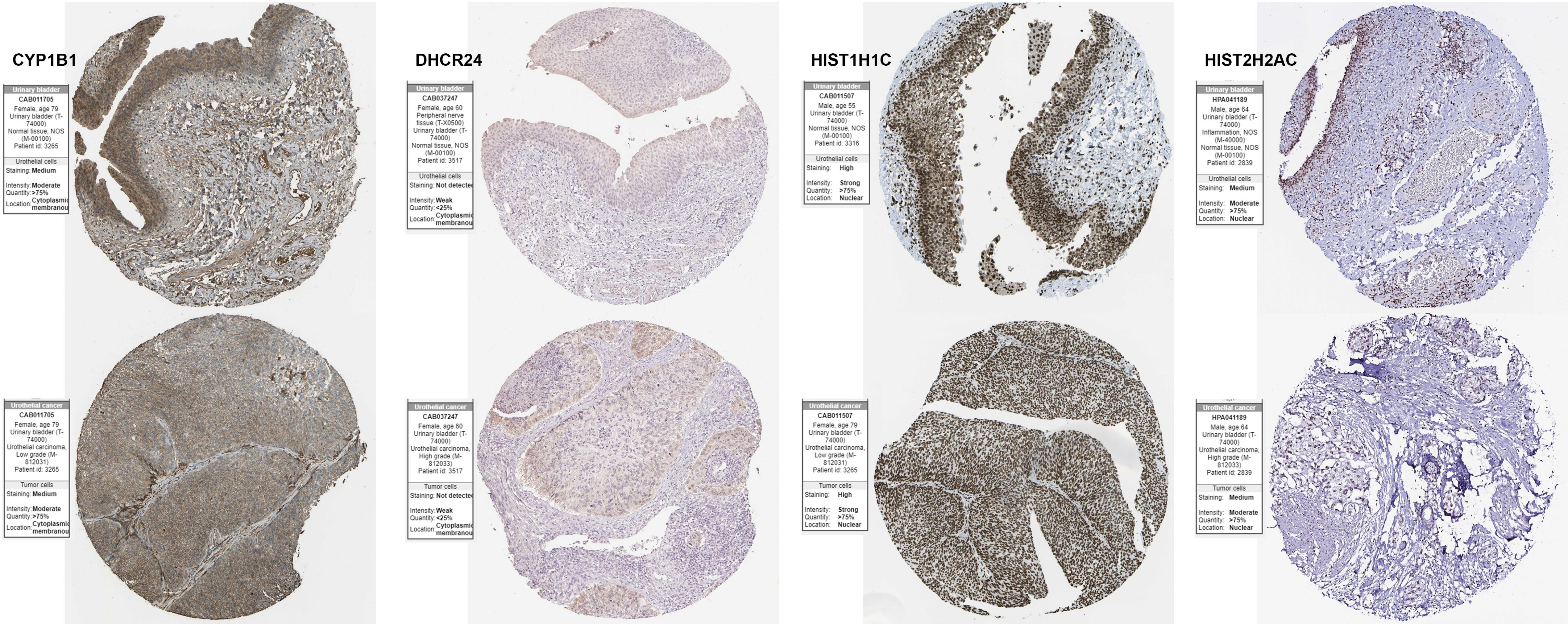

CYP1B1
Urinary bladder
CAB011705
Female, age 79
Urinary bladder (T-74000)
Normal tissue, NOS (M-00100)
Patient id: 3265
Urothelial cells
Staining: Medium
Intensity: Moderate
Quantity: >75%
Location: Cytoplasmic membranou
Urothelial cancer
CAB011705
Female, age 79
Urinary bladder (T-74000)
Urothelial carcinoma, Low grade (M-812031)
Patient id: 3265
Tumor cells
Staining: Medium
Intensity: Moderate
Quantity: >75%
Location: Cytoplasmic membranou
DHCR24
Urinary bladder
CAB037247
Female, age 60
Peripheral nerve tissue (T-X0500)
Urinary bladder (T-74000)
Normal tissue, NOS (M-00100)
Patient id: 3517
Urothelial cells
Staining: Not detecte
Intensity: Weak
Quantity: <25%
Location: Cytoplasmic membranou
Urothelial cancer
CAB037247
Female, age 60
Urinary bladder (T-74000)
Urothelial carcinoma, High grade (M-812033)
Patient id: 3517
Tumor cells
Staining: Not detecte
Intensity: Weak
Quantity: <25%
Location: Cytoplasmic membranou
HIST1H1C
Urinary bladder
CAB011507
Male, age 55
Urinary bladder (T-74000)
Normal tissue, NOS (M-00100)
Patient id: 3316
Urothelial cells
Staining: High
Intensity: Strong
Quantity: >75%
Location: Nuclear
Urothelial cancer
CAB011507
Female, age 79
Urinary bladder (T-74000)
Urothelial carcinoma, Low grade (M-812031)
Patient id: 3265
Tumor cells
Staining: High
Intensity: Strong
Quantity: >75%
Location: Nuclear
HIST2H2AC
Urinary bladder
HPA041189
Male, age 64
Urinary bladder (T-74000)
Inflammation, NOS (M-40000)
Normal tissue, NOS (M-00100)
Patient id: 2839
Urothelial cells
Staining: Medium
Intensity: Moderate
Quantity: >75%
Location: Nuclear
Urothelial cancer
HPA041189
Male, age 64
Urinary bladder (T-74000)
Urothelial carcinoma, High grade (M-812033)
Patient id: 2839
Tumor cells
Staining: Medium
Intensity: Moderate
Quantity: >75%
Location: Nuclear

FIGURE S2

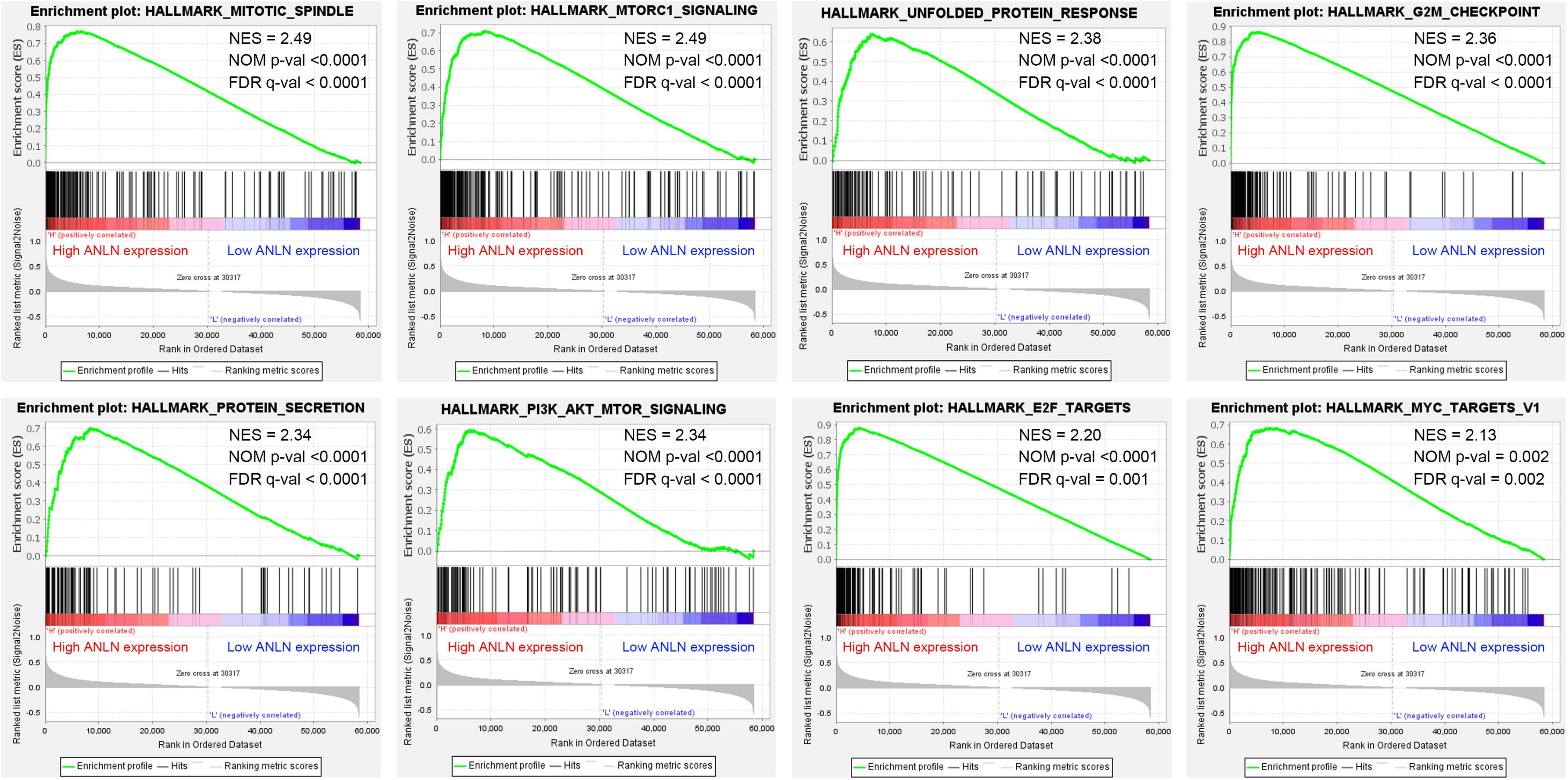

Enrichment plot: HALLMARK_MITOTIC_SPINDLE
NES = 2.49
NOM p-val <0.0001
FDR q-val < 0.0001
Enrichment plot: HALLMARK_MTORC1_SIGNALING
NES = 2.49
NOM p-val <0.0001
FDR q-val < 0.0001
HALLMARK_UNFOLDED_PROTEIN_RESPONSE
NES = 2.38
NOM p-val <0.0001
FDR q-val < 0.0001
Enrichment plot: HALLMARK_G2M_CHECKPOINT
NES = 2.36
NOM p-val <0.0001
FDR q-val < 0.0001
Enrichment plot: HALLMARK_PROTEIN_SECRETION
NES = 2.34
NOM p-val <0.0001
FDR q-val < 0.0001
HALLMARK_PI3K_AKT_MTOR_SIGNALING
NES = 2.34
NOM p-val <0.0001
FDR q-val < 0.0001
Enrichment plot: HALLMARK_E2F_TARGETS
NES = 2.20
NOM p-val <0.0001
FDR q-val = 0.001
Enrichment plot: HALLMARK_MYC_TARGETS_V1
NES = 2.13
NOM p-val = 0.002
FDR q-val = 0.002
Enrichment score (ES)
Ranked list metric (Signal2Noise)
'H' (positively correlated)
High ANLN expression
Low ANLN expression
Zero cross at 30317
'L' (negatively correlated)
Rank in Ordered Dataset
Enrichment profile — Hits — Ranking metric scores

FIGURE S3

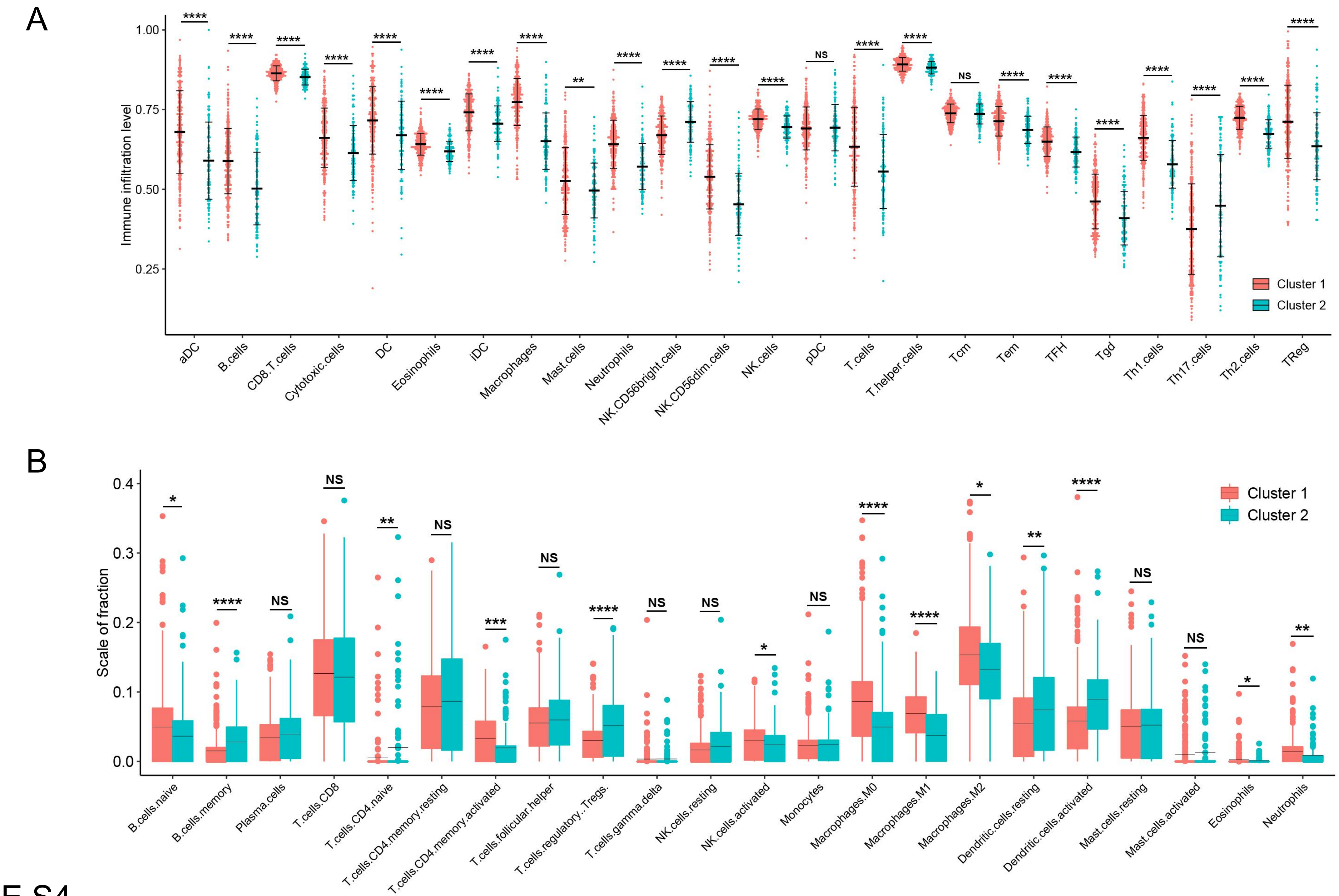
A
Immune infiltration level
1.00
0.75
0.50
0.25
Cluster 1
Cluster 2
aDC
B.cells
CD8.T.cells
Cytotoxic.cells
DC
Eosinophils
iDC
Macrophages
Mast.cells
Neutrophils
NK.CD56bright.cells
NK.CD56dim.cells
NK.cells
pDC
T.cells
T.helper.cells
Tcm
Tem
TFH
Tgd
Th1.cells
Th17.cells
Th2.cells
TReg
B
Scale of fraction
0.4
0.3
0.2
0.1
0.0
Cluster 1
Cluster 2
B.cells.naive
B.cells.memory
Plasma.cells
T.cells.CD8
T.cells.CD4.naive
T.cells.CD4.memory.resting
T.cells.CD4.memory.activated
T.cells.follicular.helper
T.cells.regulatory..Tregs.
T.cells.gamma.delta
NK.cells.resting
NK.cells.activated
Monocytes
Macrophages.M0
Macrophages.M1
Macrophages.M2
Dendritic.cells.resting
Dendritic.cells.activated
Mast.cells.resting
Mast.cells.activated
Eosinophils
Neutrophils

FIGURE S4

A

P-value = 0.5738

Event	Validation	Training
High grade	189	189
Low Grade	8	12

P-value = 0.3527

Event	Validation	Training
Female	48	58
Male	151	144

P-value = 0.4556

Event	Validation	Training
Stage II	60	67
Stage III	72	67
Stage IV	65	68

P-value = 0.9052

Event	Validation	Training
Cluster1	140	140
Cluster2	59	62

P-value = 0.3879

Event	Validation	Training
T0	0	1
T1	1	0
T2	20	17
T2a	10	15
T2b	29	26
T3	17	26
T3a	29	40
T3b	43	37
T4	7	3
T4a	24	19
T4b	4	1

P-value = 0.3616

Event	Validation	Training
N0	116	117
N1	18	28
N2	41	34
N3	3	4
NX	20	15

P-value = 0.9773

Event	Validation	Training
M0	97	96
M1	6	5
M2	95	100

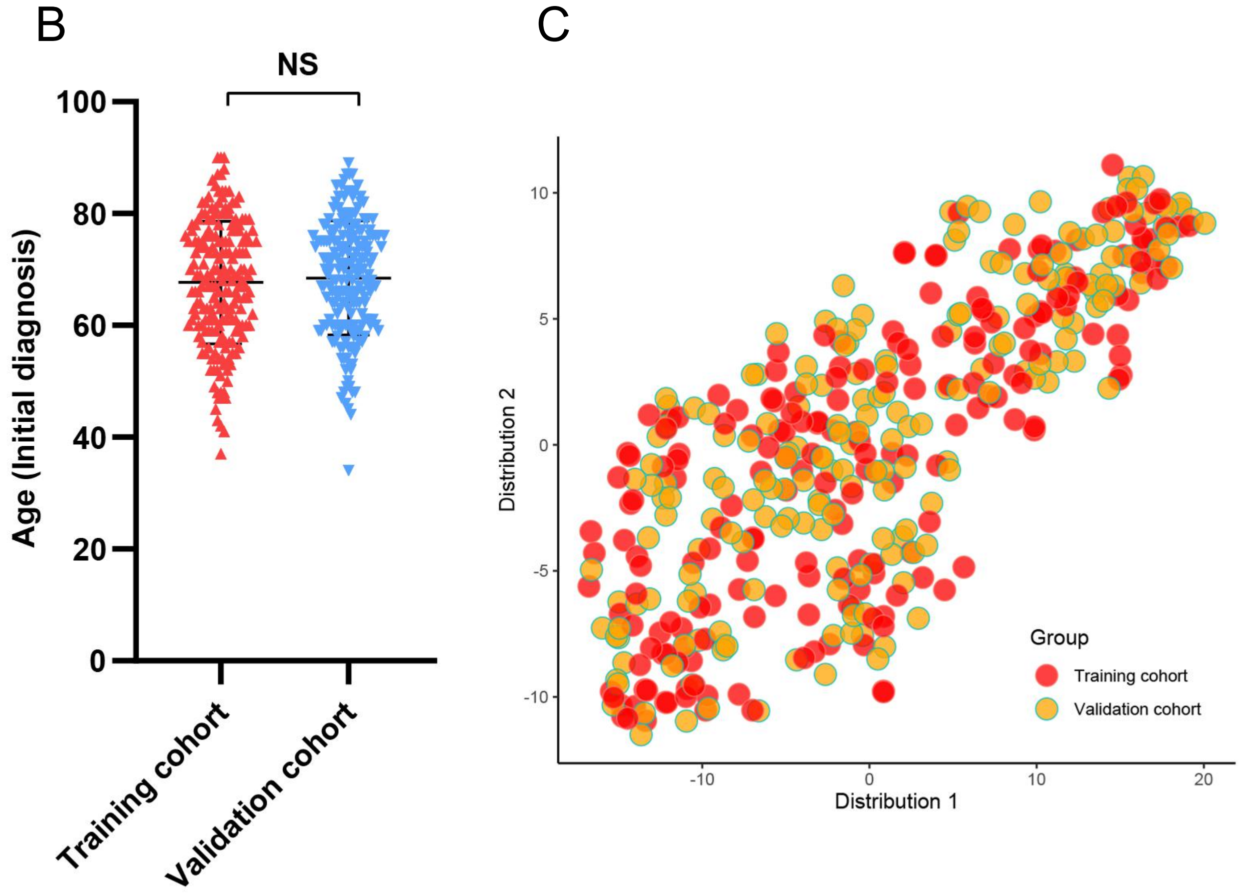

FIGURE S5

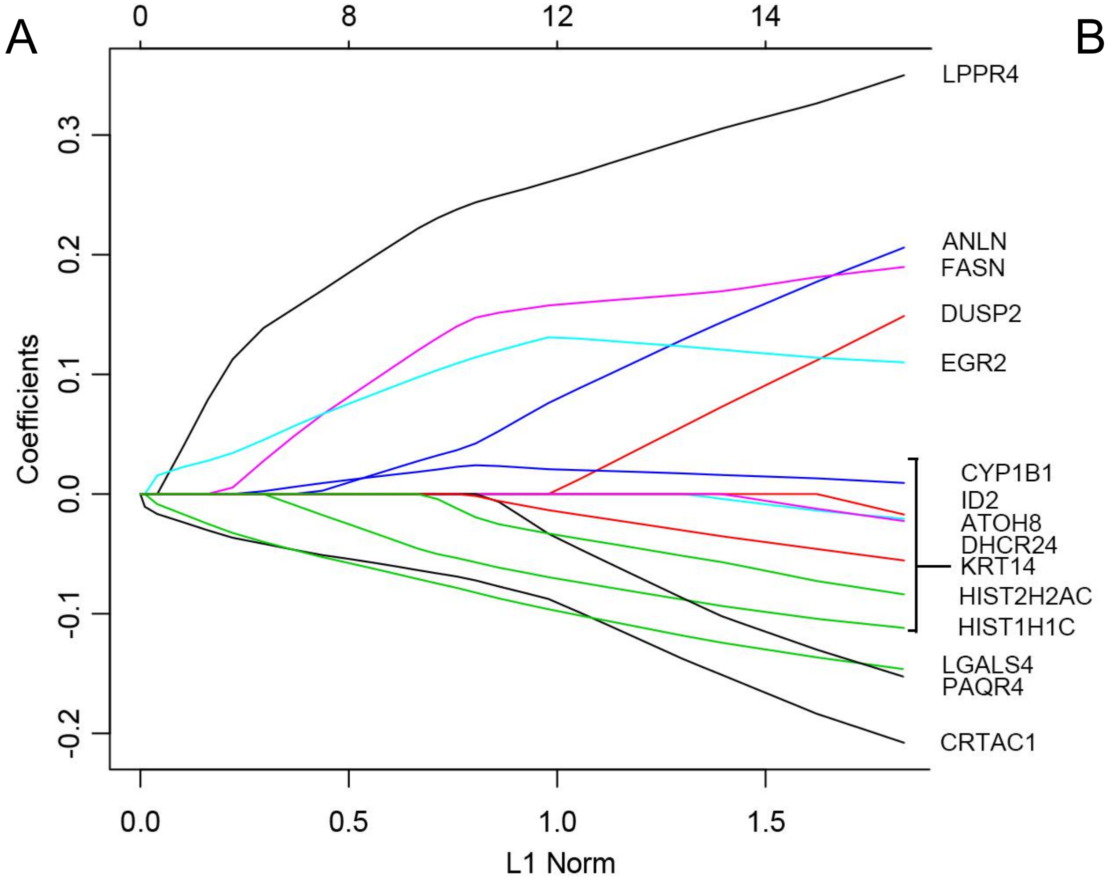
A
0
8
12
14
LPPR4
ANLN
FASN
DUSP2
EGR2
CYP1B1
ID2
ATOH8
DHCR24
KRT14
HIST2H2AC
HIST1H1C
LGALS4
PAQR4
CRTAC1
Coefficients
0.3
0.2
0.1
0.0
-0.1
-0.2
0.0
0.5
1.0
1.5
L1 Norm
B
15 15 15 15 14 14 13 12 11 8 8 6 4 0
Partial Likelihood Deviance
11.2
11.0
10.8
10.6
10.4
-6
-5
-4
-3
-2
Log(λ)

FIGURE S6

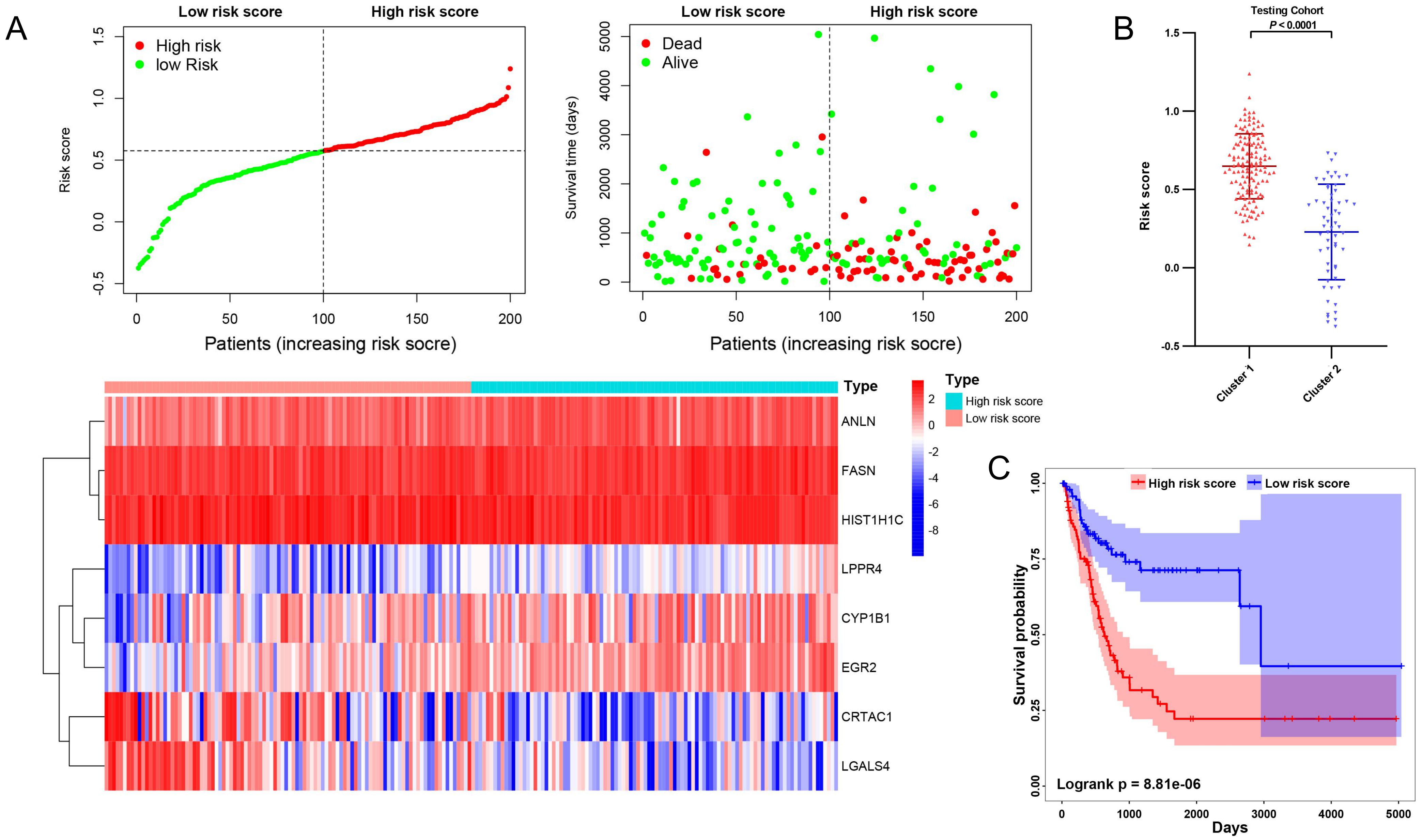
A
Low risk score
High risk score
High risk
low Risk
Risk score
Patients (increasing risk socre)
Dead
Alive
Survival time (days)
B
Testing Cohort
P < 0.0001
Cluster 1
Cluster 2
Type
High risk score
Low risk score
ANLN
FASN
HIST1H1C
LPPR4
CYP1B1
EGR2
CRTAC1
LGALS4
C
High risk score
Low risk score
Survival probability
Logrank p = 8.81e-06
Days

FIGURE S7

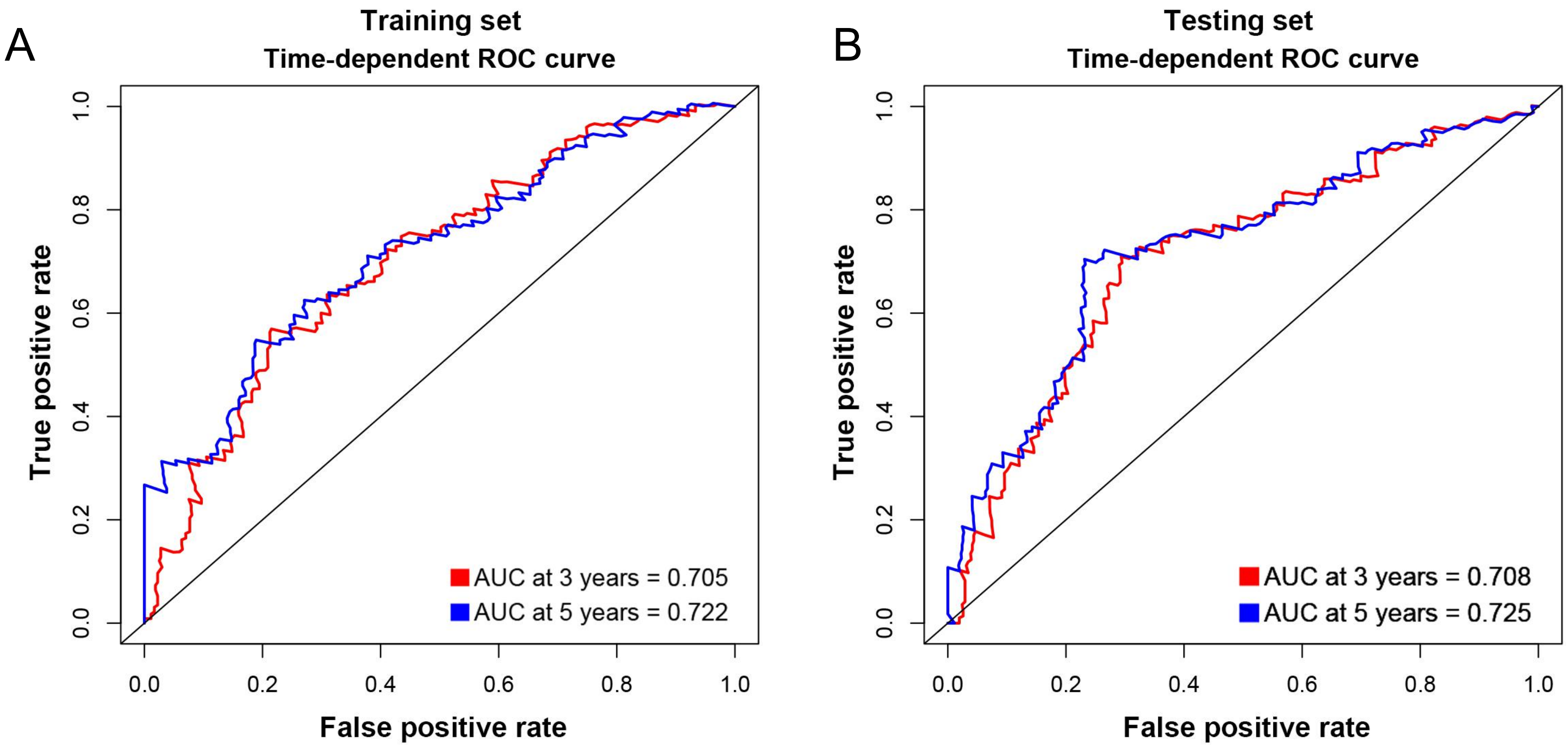
A
Training set
Time-dependent ROC curve
True positive rate
False positive rate
0.0
0.2
0.4
0.6
0.8
1.0
AUC at 3 years = 0.705
AUC at 5 years = 0.722
B
Testing set
Time-dependent ROC curve
True positive rate
False positive rate
AUC at 3 years = 0.708
AUC at 5 years = 0.725

FIGURE S8

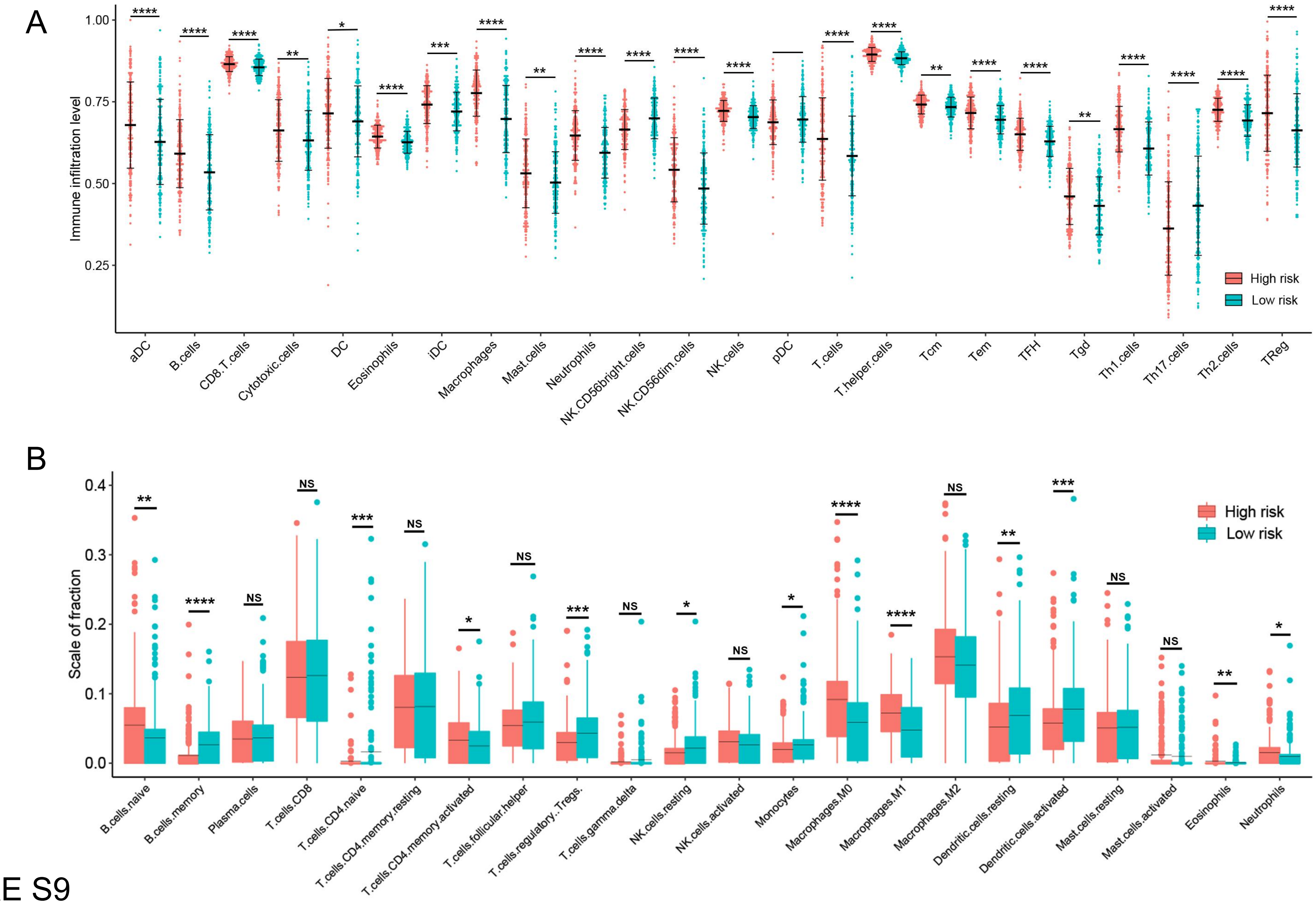
A
Immune infiltration level
1.00
0.75
0.50
0.25
High risk
Low risk
aDC
B.cells
CD8.T.cells
Cytotoxic.cells
DC
Eosinophils
iDC
Macrophages
Mast.cells
Neutrophils
NK.CD56bright.cells
NK.CD56dim.cells
NK.cells
pDC
T.cells
T.helper.cells
Tcm
Tem
TFH
Tgd
Th1.cells
Th17.cells
Th2.cells
TReg
B
Scale of fraction
0.4
0.3
0.2
0.1
0.0
High risk
Low risk
B.cells.naive
B.cells.memory
Plasma.cells
T.cells.CD8
T.cells.CD4.naive
T.cells.CD4.memory.resting
T.cells.CD4.memory.activated
T.cells.follicular.helper
T.cells.regulatory..Tregs.
T.cells.gamma.delta
NK.cells.resting
NK.cells.activated
Monocytes
Macrophages.M0
Macrophages.M1
Macrophages.M2
Dendritic.cells.resting
Dendritic.cells.activated
Mast.cells.resting
Mast.cells.activated
Eosinophils
Neutrophils

FIGURE S9

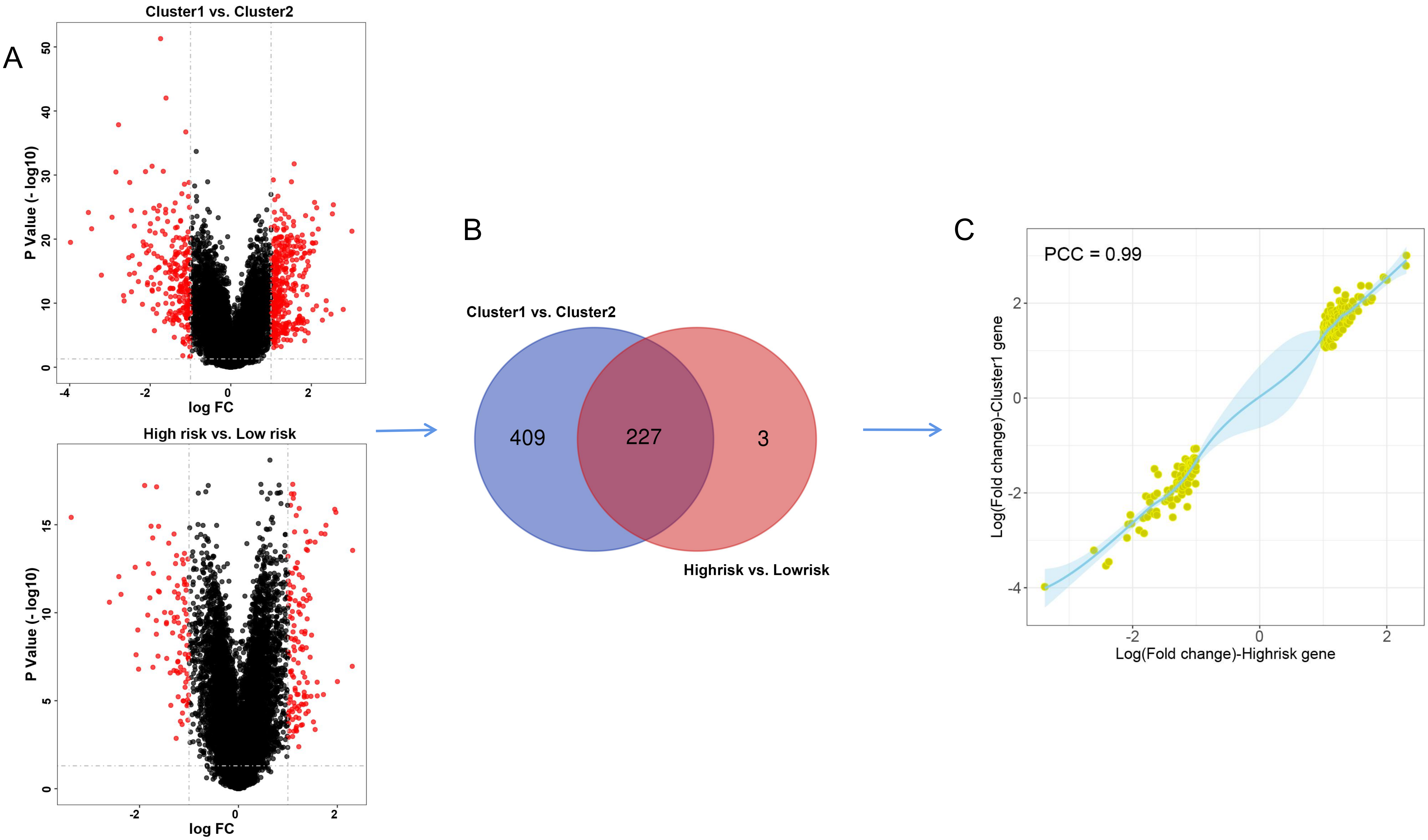
A
Cluster1 vs. Cluster2
P Value (- log10)
log FC
High risk vs. Low risk
P Value (- log10)
log FC
B
Cluster1 vs. Cluster2
409
227
3
Highrisk vs. Lowrisk
C
PCC = 0.99
Log(Fold change)-Cluster1 gene
Log(Fold change)-Highrisk gene

FIGURE S10
